# ZEB1 promotes chemoimmunotherapy resistance in pancreatic cancer models by downregulating chromatin acetylation of *CXCL16*

**DOI:** 10.1172/JCI195970

**Published:** 2025-09-09

**Authors:** Shaobo Zhang, Yumeng Hu, Zhijun Zhou, Gaoyuan Lv, Chenze Zhang, Yuanyuan Guo, Fangxia Wang, Yuxin Ye, Haoran Qi, Hui Zhang, Wenming Wu, Min Li, Mingyang Liu

**Affiliations:** 1 State Key Laboratory of Molecular Oncology, National Cancer Center/National Clinical Research Center for Cancer/Cancer Hospital, Chinese Academy of Medical Sciences and Peking Union Medical College, Beijing, China.; 2 Department of Medicine and; 3 Department of Surgery, University of Oklahoma Health Sciences Center, Oklahoma City, Oklahoma, USA.; 4 National Key Laboratory of Efficacy and Mechanism on Chinese Medicine for Metabolic Diseases, Beijing Research Institute of Chinese Medicine, Beijing University of Chinese Medicine, Beijing, China.; 5 Department of Pathology and; 6 Department of General Surgery, Peking Union Medical College Hospital, Chinese Academy of Medical Sciences and Peking Union Medical College, Beijing, China.

**Keywords:** Cell biology, Oncology, Cancer immunotherapy, Epigenetics, Neutrophils

## Abstract

Pancreatic cancer (PC) is notoriously resistant to both chemotherapy and immunotherapy, presenting a major therapeutic challenge. Epigenetic modifications play a critical role in PC progression, yet their contribution to chemoimmunotherapy resistance remains poorly understood. Here, we identified the transcription factor ZEB1 as a critical driver of chemoimmunotherapy resistance in PC. *ZEB1* knockdown synergized with gemcitabine and anti–PD-1 therapy, markedly suppressed PC growth, and prolonged survival in vivo. Single-cell and spatial transcriptomics revealed that ZEB1 ablation promoted tumor pyroptosis by recruiting and activating GZMA^+^CD8^+^ T cells in the tumor core through epigenetic upregulation of *CXCL16*. Meanwhile, ZEB1 blockade attenuates CD44^+^ neutrophil–induced CD8^+^ T cell exhaustion by reducing tumor-derived SPP1 secretion, which otherwise promotes exhaustion through activation of the PD-L1/PD-1 pathway. Clinically, high ZEB1 expression correlated with chemoresistance, immunosuppression, and diminished CXCL16 levels in patients with PC. Importantly, the epigenetic inhibitor mocetinostat (targeting ZEB1) potentiated the efficacy of chemoimmunotherapy, including anti–PD-1 and CAR T therapies, in patient-derived organoids, xenografts, and orthotopic models. Our study unveils ZEB1 as a master epigenetic regulator of chemoimmunotherapy resistance and proposes its targeting as a transformative strategy for PC treatment.

## Abstract 2

Blocking ZEB1 reverses chemoimmunotherapy resistance in pancreatic cancer by boosting CD8+ T cells, reducing neutrophils, and enhancing tumor-killing immune responses.

## Graphical abstract

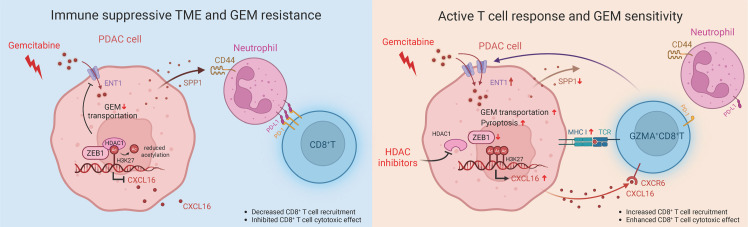

## Introduction

Pancreatic cancer (PC) is one of the most lethal cancers and was predicted to become the second leading cause of cancer-related death within this decade (1). Only a small proportion of patients with PC would benefit from targeted therapy and immunotherapy (2–4). Intratumor heterogeneity, driven by the unique genomic alterations and the immunosuppressive subpopulation of immune cells and stromal cells in the tumor microenvironment, leads to immune escape and treatment resistance in PC (5–7). Combination therapies may hold promise for improving treatment outcomes. Unfortunately, chemotherapy did not increase the effectiveness of immunotherapy in PC. Emerging evidence indicates that chemotherapy resistance has the potential to facilitate immune evasion via upregulation of immunosuppressive molecules such as CD47, PD-L1, and PGE_2_ through metabolic or oncogenic pathway reprogramming (8, 9). It fostered an immunosuppressive microenvironment in PC (10, 11). The tumor microenvironment is critical in driving the malignant phenotypes and treatment resistance (12–16). Dissecting the mechanisms through which the reprogrammed microenvironment grants PC cells the ability to escape the cytotoxic effect of chemotherapy and immunotherapy is key to fostering potential therapeutic strategies, especially combination therapy.

Cellular pyroptosis is a form of inflammatory cell death triggered by pore-forming amino-terminal fragments generated through cleavage of gasdermin family proteins. It is characterized by cell membrane perforation, activation of inflammasomes, and release of proinflammatory cytokines such as IL-1β and IL-18. In recent years, CD8^+^ T cells, as key effector cells of the cytotoxic immune response, have been shown to exert antitumor effects by inducing tumor cell pyroptosis, primarily through release of granzyme A (GZMA) and GZMB. Mechanistically, GZMA induces pyroptosis in target cells by cleaving gasdermin B (GSDMB) (17), while GZMB not only activates caspase-3 in target cells (18), but also directly cleaves GSDME at the same site as caspase-3, thereby triggering pyroptosis (19). Meanwhile, gemcitabine, a nucleoside analog, has been found to indirectly promote pyroptosis by modulating mitochondrial ROS and activating the caspase-3/GSDME pathway, in addition to its direct inhibitory effects on tumor cell proliferation (20). These findings provide a potential theoretical basis for combining immunotherapy with chemotherapy.

Epigenetic modification, such as DNA and histone modification, profoundly affects the tumor immune microenvironment (TIME) by dynamically modifying gene expression in the tumor microenvironment. Through inhibition of DNA methylation, suppression of immune-related genes can be reversed, leading to an increase in the number and function of tumor-infiltrating CD8^+^ T cells and thereby restoring immune function (21). Epigenetic modification induced cancer immune evasion by decreasing tumor immunogenicity, a critical factor associated with neoantigen quality and its presentation (22, 23). Histone acetylation modulates chromatin accessibility, which plays a pivotal role in cancer immune evasion (24–29).

Histone deacetylases (HDACs) are a group of enzymes that remove acetyl groups from histones. HDAC inhibition increases the sensitivity of chemotherapy and suppresses PC progression by blocking phenotypic transformation of fibroblasts in preclinical models (30, 31). HDAC1 is identified as a marker of poor immune checkpoint blockade (ICB) response in hepatocellular carcinoma (32); its inhibition enhances CD8^+^ T cell activity and improves immunotherapy efficacy in lung and colorectal cancers (33). However, its role in PC remains unclear. A phase II clinical trial showed that HDAC inhibitor had a synergistic effect when combined with anti–PD-1 immunotherapy and anti-VEGF antibody in patients with proficient mismatch-repair/microsatellite-stable (pMMR/MSS) colorectal cancer, who are deemed resistant to immunotherapy (34). However, whether combining immunotherapy, chemotherapy, and HDAC inhibitors would provide synergistic efficacy in PC remains to be determined. While zinc finger E-box binding homeobox 1 (ZEB1) is known to play critical roles in chemoresistance and cellular plasticity (35, 36), its contribution to chemoimmunotherapy resistance, particularly through regulation of HDAC-associated chromatin accessibility and immune microenvironment reprogramming, remains poorly understood.

In this study, we identified that knocking down *ZEB1* substantially inhibited PC progression and enhanced chemoimmunotherapy response in vivo through enhancing CD8^+^ T cell–induced pyroptosis and inhibiting crosstalk between CD8^+^ T cells and neutrophils in PC. Treatment with mocetinostat (an epigenetic reprogramming inhibitor of ZEB1) synergizes with gemcitabine and anti–PD-1, enhancing the efficacy of chemotherapy and immunotherapy in an allograft mouse model, patient-derived organoid models, and patient-derived xenograft mouse models.

## Results

### ZEB1 promotes chemoimmunotherapy resistance in PC.

Given that patients with PC are resistant to chemoimmunotherapy, we established 2 human PC stable cell lines (AsPC-1-R and MIA PaCa-2-R) that are resistant to gemcitabine and inactivate CD8^+^ T cells (Supplemental Figure 1, A–F; supplemental material available online with this article; https://doi.org/10.1172/JCI195970DS1). To investigate the underlying mechanism, we performed RNA sequencing in WT and chemoimmunotherapy-resistant PC cell lines. The upregulated transcription factors (TFs) in chemoimmunotherapy-resistant PC cells were merged with TFs that were upregulated in PC tissue and HDAC-interacting TFs (Supplemental Figure 1G). We identified 6 genes and finally focused on *ZEB1*, which has been reported to promote tumor progression and migration. We found that *ZEB1* was upregulated upon gemcitabine treatment as well as in gemcitabine-resistant stable cell lines (Supplemental Figure 1, H and I). Knockdown (KD) of *ZEB1* increased the sensitivity of PC to gemcitabine and activated CD8^+^ T cells (Supplemental Figure 1, J–M).

### Targeting ZEB1 activates TIME and sensitizes PC to chemoimmunotherapy.

**Figure 1:**
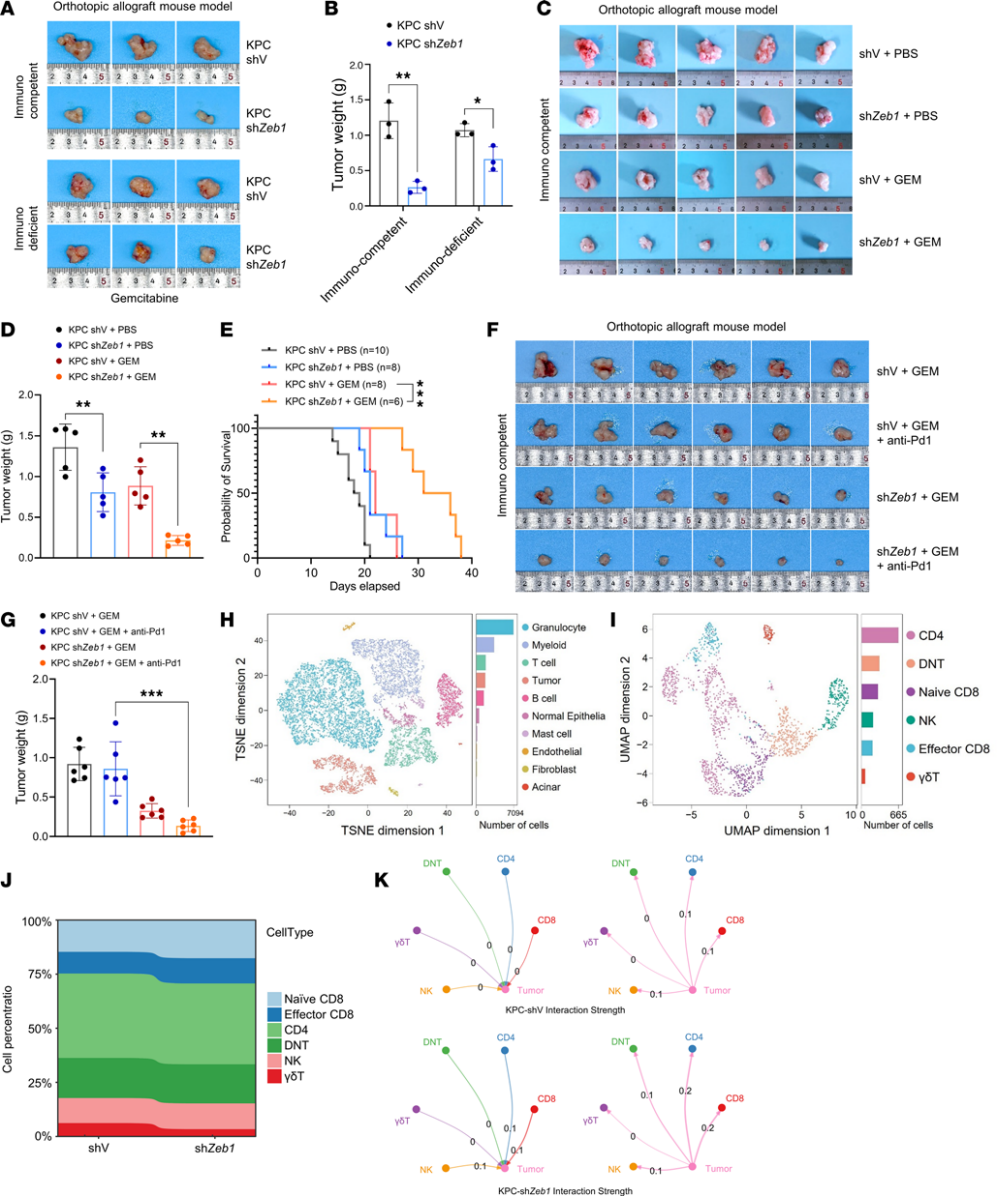
Blocking of Zeb1 enhances gemcitabine efficacy through activation of the PC immune microenvironment. (**A** and **B**) Tumor images and weight of orthotopic allograft mouse model (immunocompetent and immunodeficient) established from KPC-shV and KPC-sh*Zeb1* cells and treated with gemcitabine (GEM; 50 mg/kg) 3 times a week (*n* = 3). (**C** and **D**) Tumor images and weight of orthotopic allograft mouse model established from KPC-shV and KPC-sh*Zeb1* cells in each treatment condition (*n* = 5). (**E**) Survival of orthotopic allograft mouse model established from KPC-shV and KPC-sh*Zeb1* cells in each treatment condition (*n* = 6–10). (**F** and **G**) Tumor images and weight of orthotopic allograft mouse model established from KPC-shV and KPC-sh*Zeb1* cells and treated with gemcitabine (50 mg/kg) and anti–PD-1 (10 mg/kg) 3 times a week (*n* = 6). (**H**) Uniform manifold approximation and projection (UMAP) plot of scRNA-Seq data derived from orthotopic allograft mouse model reveals the presence of 10 distinct cell types. Cell types are distinguished by color. (**I**) UMAP plot displays the distribution and subclustering of T and NK cell subsets. TSNE, t-distributed stochastic neighbor embedding. (**J**) Stacked histogram shows the proportion of each T/NK cluster in KPC-shV and KPC-sh*Zeb1* mouse tumor tissues. (**K**) Circle plots depict the strength of cell-cell interactions between subclusters of T/NK cells and tumor cells, as identified through CellChat analysis. The edge weights and numerical values indicate the strength score of these interactions, while the direction of the arrows denotes the cell clusters responsible for signaling release and reception. *P < 0.05, **P < 0.01, ***P < 0.001, by unpaired, 2-tailed Student’s *t* test (**B**), 1-way ANOVA with Tukey’s multiple-comparison test (**D** and **G**), and log-rank test (**E**). Data are presented as mean ± SD in **B**, **D**, and **G**.

Next, we assessed the impact of *ZEB1* KD on chemoimmunotherapy sensitivity in vivo using an allograft PC mouse model (Supplemental Figure 1N). To investigate whether knocking down *ZEB1* synergizes with chemoimmunotherapy through TIME, we orthotopically inoculated control (KPC-shV) and *ZEB1*-KD KPC (KPC-sh*ZEB1*) cells into immunocompetent and immunodeficient mice under treatment with gemcitabine. The results showed that ZEB1 inhibition induced more dramatic tumor regression in immunocompetent mice (Figure 1, A and B). Inhibition of ZEB1 notably enhanced the tumor-suppressive effect of gemcitabine and prolonged overall survival (OS) in immunocompetent mice (Figure 1, C–E, and Supplemental Figure 1, O and P). Further experiments confirmed that *ZEB1* KD in combination with gemcitabine and anti–PD-1 therapy resulted in superior tumor suppression (Figure 1, F and G). To further investigate the function of ZEB1 on TIME, we performed single-cell RNA-Seq (scRNA-Seq) using the tumor tissue collected from mice allografted with KPC-shV or KPC-sh*ZEB1* cells treated with gemcitabine shown in Figure 1, C, H, and I. Compared with control, *ZEB1* KD substantially increased the proportion of total T cells and CD8^+^ T cells (Figure 1J, Supplemental Figure 1Q, and Supplemental Figure 2, A and B). These findings were validated by IHC staining and flow cytometry analysis (Supplemental Figure 1, R and S). Ligand-receptor pair communication analysis revealed enhanced interaction between PC cells and T cells following *ZEB1* KD (Figure 1K). These findings indicated that blocking ZEB1 enhanced chemoimmunotherapy through activation of CD8^+^ T cells in vivo.

### GZMA^+^CD8^+^ T cells are enriched in ZEB1-KD tumors with gemcitabine treatment.

**Figure 2:**
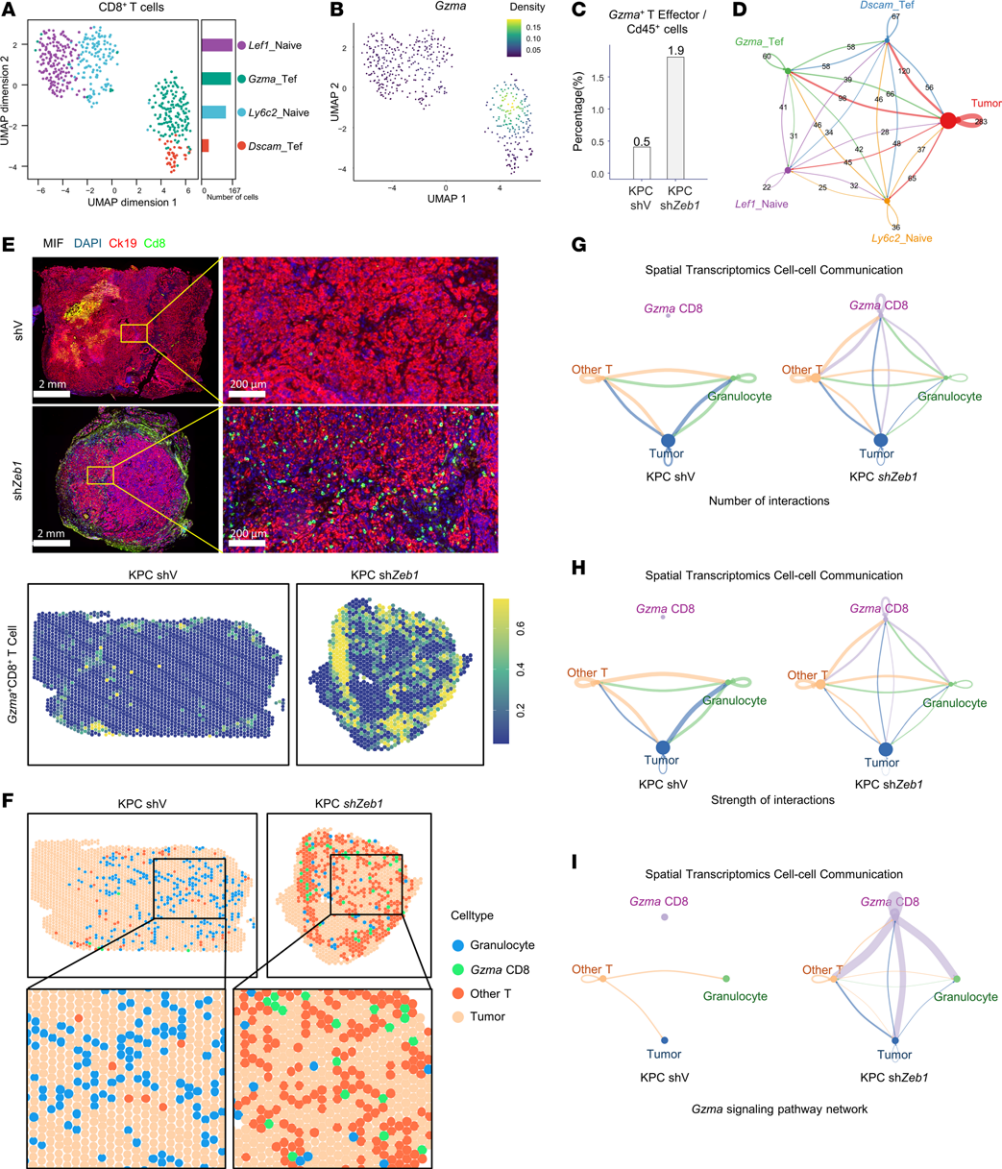
Increased infiltration of *Gzma*^+^ CD8^+^ T cells in tumor tissue with *Zeb1* KD. (**A**) UMAP reveals that CD8^+^ T cells can be classified into 4 distinct major subtypes. Tef, T effector. (**B**) Density plot shows the expression of the *Gzma* gene, with brighter colors indicating higher expression. *Gzma* is mainly expressed in the *Gzma*^+^ effector CD8^+^ T cell subset. (**C**) Percentage of *Gzma*^+^ effector CD8^+^ T cells within the total Cd45^+^ population in the KPC-shV and KPC-sh*Zeb1* groups. (**D**) Number of inferred significant ligand-receptor (LR) pairs between any 2 cell types based on single-cell analysis data. (**E**) Top panels: MIF of mouse tumor tissues. Scale bars: 2 mm (left) and 200 μm (right). Bottom panels: Marker gene set scores for CD8^+^ T cells based on spatial transcriptomics data. Brighter colors indicate higher scores, suggesting a greater abundance of CD8^+^ T cells in those regions. (**F**) Spatial transcriptome sequencing displays the distribution of 4 major annotated cell types in the control and experimental groups. (**G** and **H**) Circle plots show the number and the strength score of LR among 4 cell types across 2 groups, based on spatial transcriptome data. (**I**) Specifically, the interaction strength of GZMA-related LR pairs is dramatically increased in the KPC-sh*Zeb1* group.

To identify the specific functional subtype of CD8^+^ T cells that was associated with ZEB1, we further clustered CD8^+^ T cells into LEF1–naive, LY6C2–naive, GZMA–effector, and DSCAM–effector T cells based on gene signatures (Figure 2A). Of these 4 subsets of CD8^+^ T cells, GZMA–effector T cells (GZMA^+^CD8^+^ T cells), which constituted the largest group of cytotoxic effector T cells (cytotoxic T lymphocytes [CTLs]), were increased by 3.8-fold after *ZEB1* KD (Figure 2, B–D). This subset was the only one that prominently expressed *Gzma* (Figure 2B and Supplemental Figure 2, C–E), whose role as a cytotoxic mediator in killing tumor cells has been widely reported (17, 37, 38). To further evaluate the role of GZMA^+^CD8^+^ T cells in PC, we performed spatial transcriptomics and multiplex IHC (mIHC) on the same tumor tissues as used for scRNA-Seq. We determined that the percentage of GZMA^+^CD8^+^ T cells was remarkably increased, especially in the core region of tumor tissues, with *ZEB1* KD (Figure 2, E and F). Subsequent intercellular communication analysis revealed that compared with the other 3 subtypes of CD8^+^ T cells, GZMA^+^CD8^+^ T cells had the strongest interaction with tumor cells upon ZEB1 inhibition (Figure 2, G–I). These findings demonstrated that GZMA^+^CD8^+^ T cells were the main mediators of the immune response triggered by ZEB1 inhibition in PC.

### ZEB1 inhibition enhances the anticancer response of CD8^+^ T cells and CAR T cell therapy.

**Figure 3:**
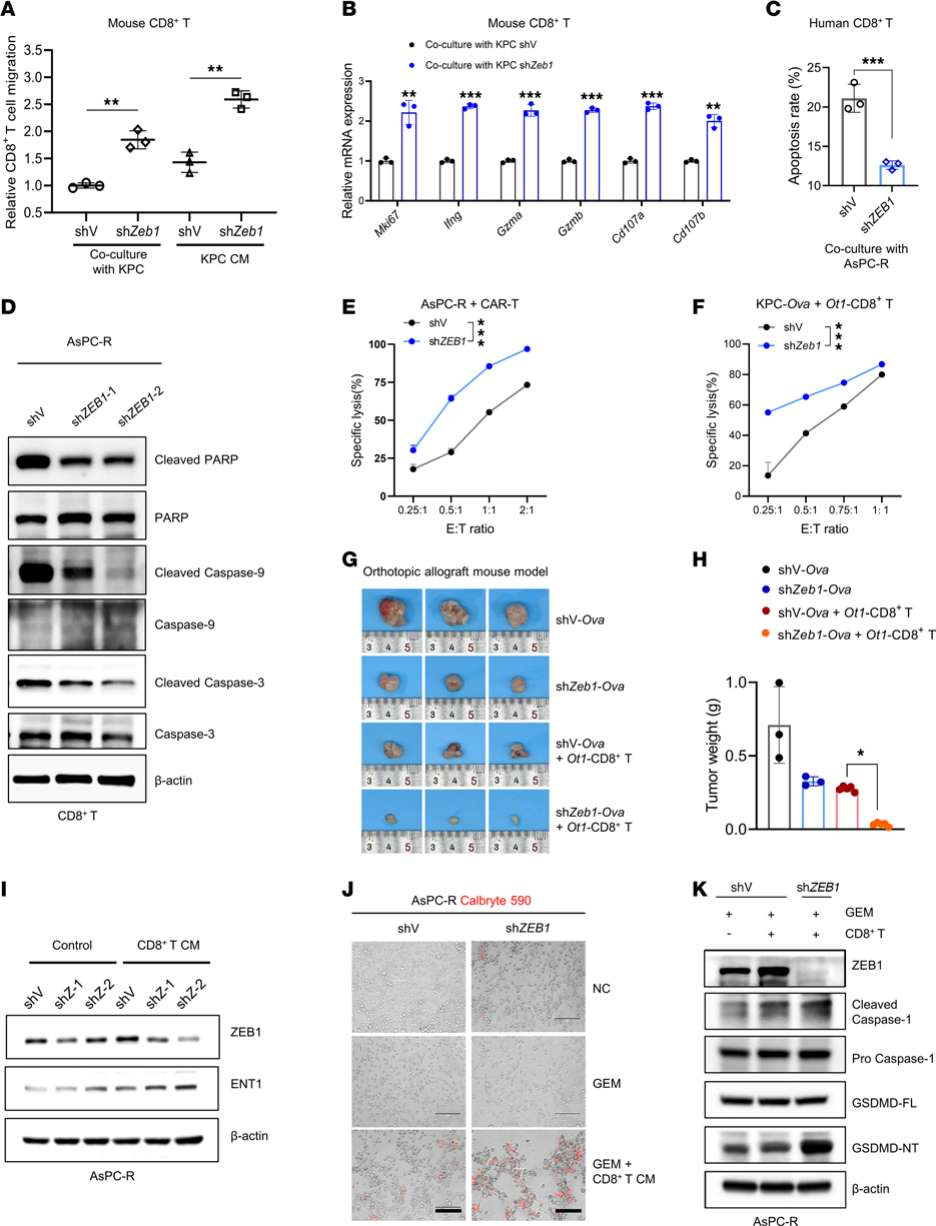
Blocking of ZEB1 enhances the antitumor activity of CD8^+^ T cells. (**A**) CD8^+^ T cell migration assay. (**B**) Detection of the level of activation markers of mouse CD8^+^ T cells by qPCR after coculturing with KPC-shV and -sh*Zeb1* cells for 48 hours. (**C**) Flow cytometry analysis of the apoptotic rate of human CD8^+^ T cells after coculturing with AsPC-R-shV and -sh*ZEB1* cells. (**D**) Western blot detection of apoptotic markers in human CD8^+^ T cells after coculturing with AsPC-R-shV, sh*ZEB1* cells. (**E**) Specific lysis of AsPC-R-shV–luciferase and AsPC-R-sh*ZEB1*–luciferase cells after coculturing with CAR T cells for 48 hours. (**F**) Detection of specific lysis of KPC-shV-*Ova*–luciferase and KPC-sh*Zeb1-Ova*–luciferase after coculturing with mouse *Ot1*-CD8^+^ T cells for 24 hours. (**G** and **H**) Tumor images and weight of orthotopic allograft mouse model established from KPC-shV-*Ova* and KPC-sh*Zeb1-Ova* cells and treated with mouse *Ot1*-CD8^+^ T cells (*n* = 3–5). (**I**) Detection of ENT1 expression in AsPC-R-shV and -sh*ZEB1* cells after treatment with CD8^+^ T cell CM for 48 hours. (**J**) Representative images of AsPC-R-shV and -sh*ZEB1* cell after treatment with gemcitabine (1,000 nM) and conditioned medium of CD8^+^ T for 48 hours (*n* = 3). Cells were labeled using the calcium ion probe Calbryte 590 (AAT Bioquest, 20700), and the red fluorescence signal represents pyroptotic cells. Scale bars: 50 μm. (**K**) Detection of pyroptotic proteins in AsPC-R-shV and -sh*ZEB1* cells after treatment with gemcitabine (1,000 nM) and coculture with CD8^+^ T cells. Data are representative of at least 3 (**A**–**F**, **I**, and **K**) independent experiments. *P < 0.05, **P < 0.01, ***P < 0.001, by unpaired, 2-tailed Student’s *t* test (**A**–**C**), 2-way ANOVA (**E** and **F**), and 1-way ANOVA with Tukey’s multiple-comparison test (**H**). Data are presented as mean ± SD in **A**–**C** and **H** and mean ± SEM in **E** and **F**.

PC is characterized by a suppressive immune microenvironment that severely limits the CTL response (39–41). In concordance with the scRNA-Seq analysis, the results of in vitro experiments illustrated that ZEB1 inhibition enhanced recruitment and activation of CD8^+^ T cells while reducing their apoptosis (Figure 3, A–D, and Supplemental Figure 3A). We evaluated whether the activated CD8^+^ T cells decreased cell viability and potentiated the gemcitabine sensitivity of PC cells, and found that CD8^+^ T cell treatment augmented gemcitabine sensitivity, which was enhanced by ZEB1 inhibition (Supplemental Figure 3, B–D). Moreover, expression of MHC-I was upregulated in ZEB1-KD PC cells (Supplemental Figure 3E). MHC-I signaling analyzed by scRNA-Seq data confirmed the strengthened interaction between CD8^+^ T cells and *ZEB1*-KD PC cells (Supplemental Figure 3F). Besides, the CAR T cell model was established to evaluate tumor lysis activity both in vitro and in vivo. Under different effector-to-target (E/T) ratios, we found that CAR T cells encountering *ZEB1*-KD AsPC-R cells had elevated lysis ability, which was also validated in the KPC-OVA/OT1-CD8^+^ T cell model (Figure 3, E and F). Furthermore, OT1-CD8^+^ T cells showed a dramatic antitumor effect in vivo when ZEB1 was knocked down in tumor tissue, highlighting the key function of ZEB1 in regulating the sensitivity of PC to CAR T cell therapy (Figure 3, G and H). Additionally, we wondered whether the recruited CD8^+^ T cells increased gemcitabine sensitivity by regulating expression of genes that are associated with gemcitabine sensitivity. Equilibrative nucleoside transporter 1 (ENT1) is a therapeutic response marker for gemcitabine. Our prior study revealed that ZEB1 induces PC gemcitabine resistance by inhibiting *ENT1* transcription (42), and thus we wondered whether CD8^+^ T cells could also modulate ENT1 expression. Treatment with conditioned medium (CM) of CD8^+^ T cells upregulated ENT1 expression and enhanced Cy5-gemcitabine accumulation in PC cells (Figure 3I and Supplemental Figure 3, G–I). Thus, in addition to their conventional cytotoxic effects, cytotoxic lymphocytes also upregulated ENT1 expression in PC cells, increasing their sensitivity to gemcitabine. Besides, a recent study showed that gemcitabine could foster pyroptosis by activating the caspase-1/GSDMD pathway in PC, and pyroptosis activation by VbP, an enzymatic activator of caspase-1, confers PC gemcitabine sensitivity (43). Meanwhile, cytotoxic lymphocytes can trigger pyroptosis in target cells (17). Accordingly, we wondered whether the recruitment of cytotoxic CD8^+^ T cells induced by *ZEB1* KD could boost gemcitabine-related pyroptosis. Gene set enrichment analysis (GSEA) of differentially expressed genes in PC cells after *ZEB1* KD revealed pyroptosis as a statistically significant pathway (Supplemental Figure 3J). We then performed a classical calcium release assay to evaluate tumor pyroptosis and found that the combination of gemcitabine and CM from CD8^+^ T cells increased calcium influx, enhancing the lethal lysis of PC cells (Figure 3J). Furthermore, KD of *ZEB1* acted synergistically with gemcitabine and CD8^+^ T cells to promote pyroptosis in PC cells (Figure 3, J and K, and Supplemental Figure 3, K and L). Collectively, these results indicate that targeting ZEB1 in PC cells synergized with gemcitabine by activating CD8^+^ T cells, thereby enhancing anticancer response and cytotoxicity.

### Blocking of ZEB1 activates CD8^+^ T cells partially by inhibiting the function of neutrophils.

**Figure 4:**
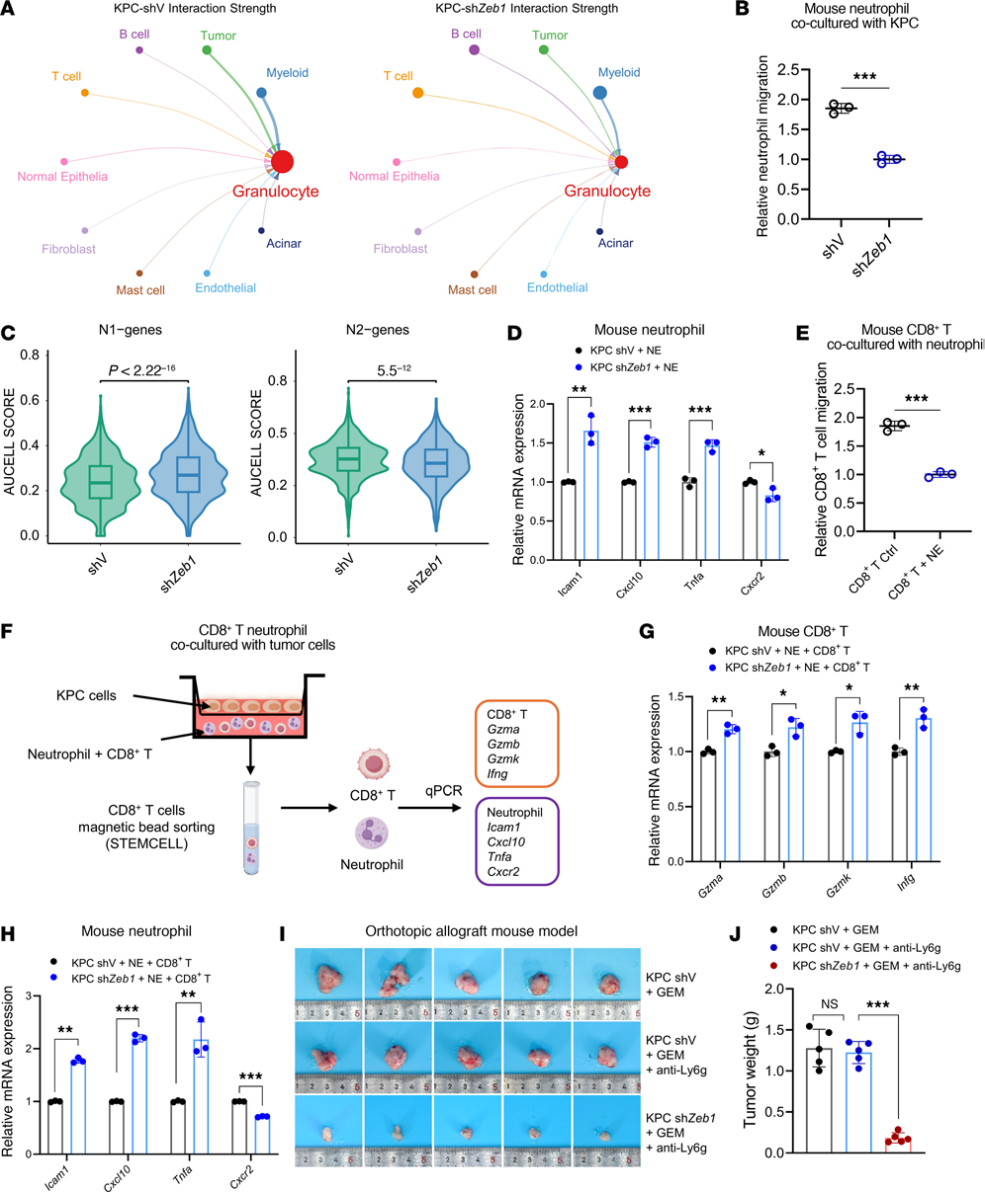
Zeb1 promotes recruitment of neutrophil and drives their polarization toward an immunosuppressive phenotype. (**A**) Circle plots compare the strengths of cell-cell interactions between granulocytes and other cell types. (**B**) Neutrophil migration assay. Relative migration of mouse neutrophils after coculture with KPC-shV and -sh*Zeb1* cells for 12 hours. (**C**) Violin plot showing AUCell scores of the N1 and N2 gene sets in neutrophils derived from the shV and sh*Zeb1* models. (**D**) Neutrophil activation. Detection of N1 polarization markers (*Icam*, *Cxcl10*, *Tnfa*) and the N2 polarization marker *Cxcr2* in neutrophils by qPCR after coculturing with KPC-shV or -sh*Zeb1* cells for 12 hours. (**E**) Relative migration of mouse CD8^+^ T cells after coculturing with neutrophils. (**F**) Schematic of a 3-cell coculture system. (**G**) CD8^+^ T cells were isolated from the 3-cell coculture systems, and levels of activation markers were detected by qPCR. (**H**) Neutrophils were isolated from the 3-cell cocultured system, and levels of N1 and N2 polarization markers were detected by qPCR. (**I** and **J**) Tumor images and weight of orthotopic allograft mouse model established from KPC-shV and -sh*Zeb1* cells and treated with gemcitabine (50 mg/kg) and anti-Ly6g (25 μg) 3 times a week (*n* = 5). Data are representative of at least 3 (**B**, **D**, **E**, **G**, and **H**) independent experiments. *P < 0.05, **P < 0.01, ***P < 0.001, by unpaired, 2-tailed Student’s *t* test (**B**, **D**, **E**, **G**, and **H**), Wilcoxon’s rank-sum test (**C**), and 1-way ANOVA with Tukey’s multiple-comparison test (**J**). Data are presented as mean ± SD in **B**, **D**, **E**, **G**, **H**, and **J**.

Our scRNA-Seq atlas analysis of TIME in vivo revealed that inhibition of ZEB1 not only increased the CD8^+^ T cell population but also decreased the proportion of granulocytes (neutrophils) and their interaction with tumor cells (Figure 4A and Supplemental Figure 1). The spatial transcriptomics and mIHC analyses indicated that there were fewer neutrophils infiltrated within the tumor’s core region when *ZEB1* was knocked down, aligning with our scRNA-Seq findings (Figure 2F). Additionally, scRNA-Seq analysis and in vitro experiments demonstrated that *ZEB1* KD inhibited neutrophil activities, including migration and polarization, thereby promoting N1-polarized neutrophil differentiation (Figure 4, B–D). Considering that neutrophils constitute a prominent immunosuppressive cell population within the tumor microenvironment (TME), leading to T cell exclusion and unresponsiveness to antigen-specific stimulation (44), we investigated whether *ZEB1* KD affects T cell response via neutrophils. Initially, we verified the suppressive effects of neutrophils on CD8^+^ T cell migration in vitro (Figure 4E). To elucidate how *ZEB1* KD in tumor cells could impair the function of CD8^+^ T cells through neutrophils, we established a coculture system with these 3 cell types and then collected them for analysis (Figure 4F). Assessment of CD8^+^ T cell and neutrophil markers revealed pronounced activation of CD8^+^ T cells and inhibition of neutrophils following *ZEB1* KD (Figure 4, G and H). Moreover, ZEB1 KD notably augmented the therapeutic efficacy of gemcitabine and anti-Ly6G combination therapies by decreasing infiltration of neutrophils, while increasing infiltration of CD8^+^ T cells (Figure 4, I and J, and Supplemental Figure 4, A–D). To pinpoint the crucial factors mediating the interaction between PC cells and neutrophils, we analyzed intercellular communications involving ligand-receptor pairs and found that the SPP1 (tumor)–CD44 (neutrophil) signal was dramatically inhibited in the *ZEB1* KD group (Supplemental Figure 4, E–G). SPP1-CD44 is critical in neutrophil recruitment and the formation of neutrophil extracellular traps (NETs). We further confirmed that KD of *ZEB1* decreased SPP1 expression both in vitro and in vivo (Supplemental Figure 4, H and I). Furthermore, SPP1 recombinant protein treatment induced a dose-dependent inhibition of neutrophil cytotoxicity (Supplemental Figure 4J). And neutrophils inhibited CD8^+^ T cell function by downregulating *PD-L1* (Supplemental Figure 4, K and L). Taken together, the results indicate that targeting ZEB1 in tumor cells effectively inhibited recruitment and polarization of neutrophils, leading to activation of CD8^+^ T cells and a synergistic antitumor effect with chemoimmunotherapy in PC.

### Inhibition of ZEB1 synergizes with chemoimmunotherapy through activation of CXCL16.

**Figure 5:**
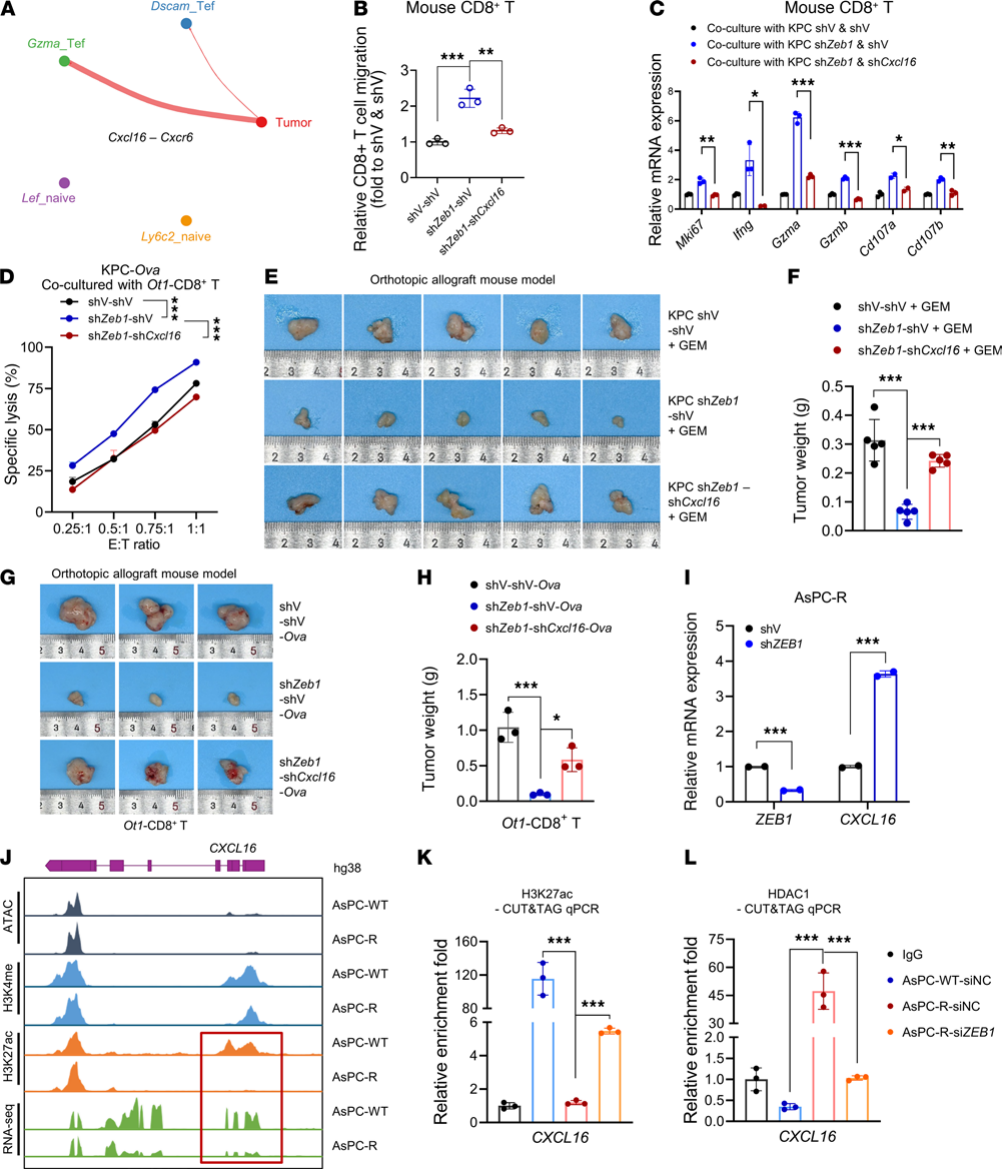
ZEB1/HDAC1 inhibits the recruitment and function of CD8^+^ T cells by epigenetically regulating CXCL16. (**A**) Circle plot showing the inferred *Cxcl16-Cxcr6* signaling network between each CD8^+^ T cell subcluster and tumor cells. Edge weights represent the strength of the interactions. (**B**) Relative migration of mouse CD8^+^ T cells, which were cocultured with KPC-shV-shV, sh*Zeb1*-shV, and KPC-sh*Zeb1*-sh*Cxcl16* cells for 48 hours. (**C**) Detection of activation markers in mouse CD8^+^ T cells, which were cocultured with tumor cells for 48 hours. (**D**) Detection of specific lysis of tumor cells after coculturing with mouse *Ot1*-CD8^+^ T cells for 24 hours. (**E** and **F**) Tumor images and weight of orthotopic allograft mouse model established from the indicated cell lines and treated with gemcitabine (50 mg/kg) 3 times a week (*n* = 5). (**G** and **H**) Tumor images and weight of orthotopic allograft mouse model established from the indicated cell lines and treated with mouse *Ot1*-CD8^+^ T cells (*n* = 3). (**I**) Relative mRNA level of *ZEB1* and *CXCL16* in AsPC-R-shV and -sh*ZEB1* cells. (**J**) ATAC-Seq, CUT&Tag-Seq of H3K27ac, CUT&Tag-Seq of H3K4me, and RNA-Seq showed changes in chromatin openness, transcriptional activity, and apparent modification levels in the *CXCL16* promoter region and gene body region. (**K** and **L**) CUT&Tag-qPCR assay of the *CXCL16* promoter region in AsPC-WT-siNC, AsPC-R-siNC, and AsPC-R-si*ZEB1* cells with antibodies against H3K27ac and HDAC1 (*n* = 3). Data are representative of at least 3 (**B**–**D**, **I**, **K**, and **L**) independent experiments. *P < 0.05, **P < 0.01, ***P < 0.001, by 1-way ANOVA with Tukey’s multiple-comparison test (**B**, **C**, **F**, **H**, **I**, **K**, and **L**) and 2-way ANOVA (**D**). Data are presented as mean ± SD in **B**, **C**, **F**, **H**, **I**, **K**, and **L**, and mean ± SEM in **D**.

To further elucidate the mechanism of ZEB1-regulated chemoimmunotherapy, we performed intercellular communication analysis using our scRNA-Seq data. We identified 64 signaling pathways that were notably upregulated in *ZEB1*-KD tumors. Among various cytokines and chemokines in KPC cells, *CXCL16* was the most markedly upregulated upon *ZEB1* KD (Figure 5A and Supplemental Figure 5A). Given that CXCR6, the specific receptor for CXCL16, is reported to be highly expressed in intratumoral CD8^+^ T cells, and that CXCR6^+^CD8^+^ T cells are critical for checkpoint blockade therapy (45–47), we hypothesized that the increased sensitivity to chemoimmunotherapy following *ZEB1* KD is attributable to the enhanced chemotaxis and activity of CD8^+^ T cells driven by elevated *CXCL16*. We confirmed the reversed correlation between ZEB1 and CXCL16 (Supplemental Figure 5, B–E). Exogenous recombinant CXCL16 increased migration and activation of CD8^+^ T cells, leading to the enhanced antitumor effect (Supplemental Figure 5, F–K). The enhancement of tumor cell recognition and CD8^+^ T cell activation in OT-1 T cells suggested a direct response to CXCL16 stimulation. To further delineate the role of the ZEB1/CXCL16 axis in CD8^+^ T cell activity and the sensitivity of PC tumors to chemoimmunotherapy or T cell therapy, we investigated CD8^+^ T cell functions. We found that *CXCL16* KD notably attenuated the migration, activation, and cytotoxicity of CD8^+^ T cells enhanced by *ZEB1* KD (Figure 5, B–D, and Supplemental Figure 5, L–N). Notably, the therapeutic benefit of chemoimmunotherapy or T cell therapy induced by ZEB1 inhibition was notably reversed by *CXCL16* KD (Figure 5, E–H, and Supplemental Figure 5, O–Q). Collectively, these results indicate that CXCL16/CXCR6 signaling, which is activated when *ZEB1* is knocked down, mediated recruitment and activation of CD8^+^ T cells, rendering PC tumors highly vulnerable to chemoimmunotherapy and T cell therapy.

### ZEB1/HDAC1 complex suppressed CD8^+^ T cells activity through epigenetic inhibition of CXCL16.

To elucidate the specific mechanism through which ZEB1 negatively regulated *CXCL16* expression to decrease response to chemoimmunotherapy, we conducted a luciferase reporter assay to assess the role of ZEB1 in *CXCL16* transcriptional regulation. The results showed that *ZEB1* KD increased *CXCL16* mRNA level but did not affect *CXCL16* promoter activity (Figure 5I and Supplemental Figure 5, R and S), suggesting that ZEB1 may regulate CXCL16 in an epigenetically dependent manner. We further investigated the modification of the *CXCL16* promoter using CUT&Tag sequencing (CUT&Tag-Seq). AsPC-1-R cells showed a clearly reduced level of H3K27ac in the *CXCL16* promoter region, while the H3K4me level only showed a slight reduction (Figure 5J). These findings imply that histone acetylation predominantly regulated CXCL16 expression in PC. CUT&Tag qPCR further confirmed a lower enrichment of H3K27ac signal in AsPC-1-R cells compared with the parental cells, while *ZEB1* KD partially restored the H3K27ac enrichment (Figure 5K). Next, we sought to elucidate the mechanism by which ZEB1 modulates H3K27 acetylation at the *CXCL16* promoter. Given that HDAC1 is a well-characterized corepressor of ZEB1 and facilitates ZEB1-mediated deacetylation of downstream targets, we performed HDAC1 CUT&Tag-qPCR in parental AsPC-1 cells and gemcitabine-resistant AsPC-1-R cells, with or without *ZEB1* KD. Strikingly, HDAC1 enrichment at the *CXCL16* promoter region was markedly elevated in gemcitabine-resistant cells, and this effect was almost completely abrogated upon *ZEB1* depletion (Figure 5L). Collectively, these findings revealed that the epigenetic modification of the *CXCL16* promoter by the HDAC1/ZEB1 complex contributed to *CXCL16* silencing in PC.

### HDAC inhibitor synergizes with chemoimmunotherapy and CAR T cell therapy in PC.

**Figure 6:**
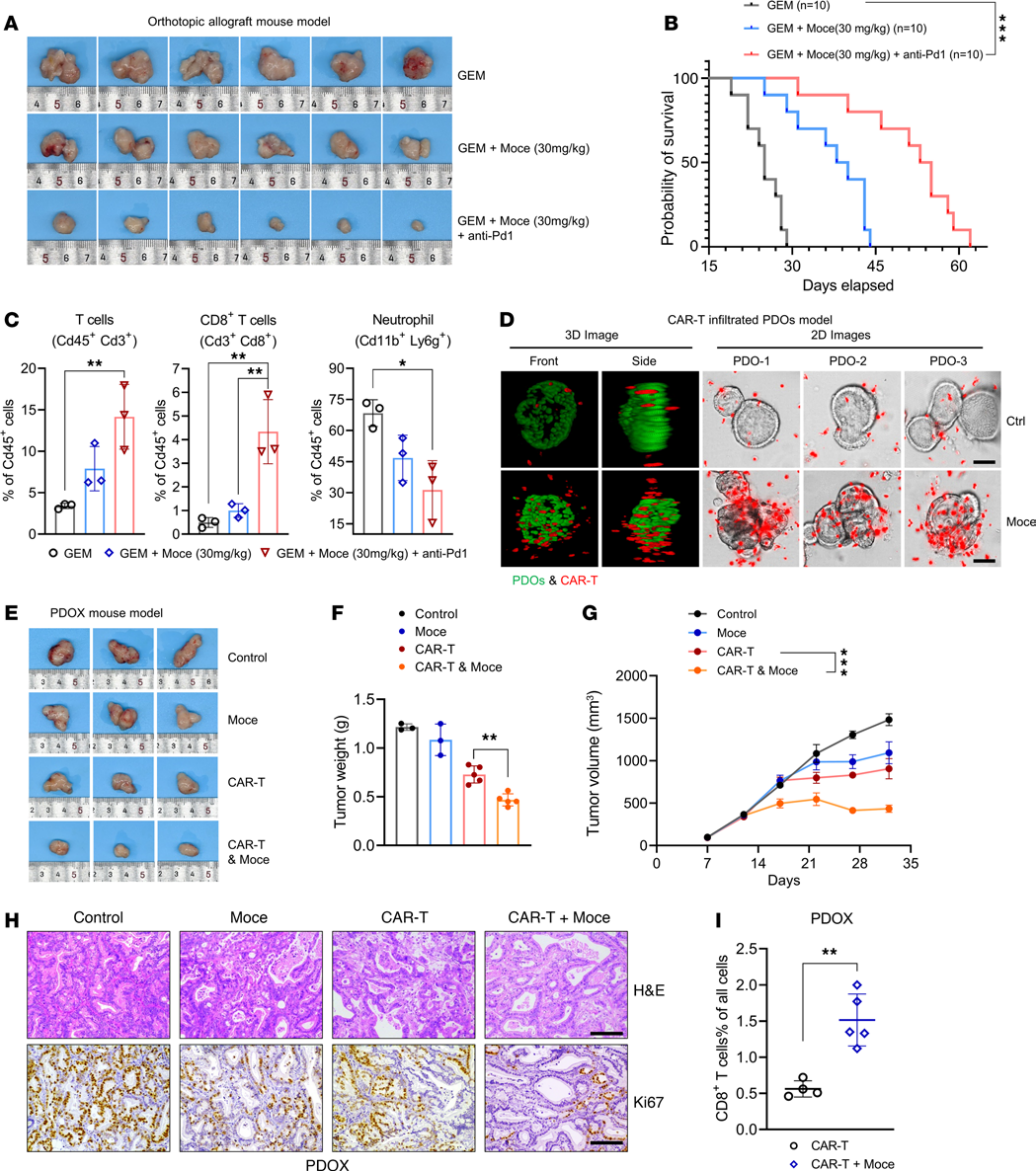
Mocetinostat enhances chemoimmunotherapy and CAR T efficacy in PC. (**A**) Tumor images of orthotopic allograft mouse model established from KPC cells in each treatment condition: gemcitabine (50 mg/kg); gemcitabine+Moce (30 mg/kg); gemcitabine+Moce (30 mg/kg) + anti–PD-1 (10 mg/kg), 3 times a week (*n* = 6). (**B**) Survival of orthotopic allograft mouse model established from KPC cells in each treatment condition (*n* = 10). (**C**) Flow cytometry analysis of the proportion of all T cells (Cd45^+^, Cd3^+^), CD8^+^ T cells (Cd3^+^, Cd8^+^), and neutrophils (Cd11b^+^, Ly6g^+^) to total Cd45^+^ cells in tumor tissue (*n* = 3). (**D**) CAR T–infiltrated PDO model: CAR T was used to infect PDOs for 24 hours after the 24 hours of Moce (500 nM) treatment of PDOs (*n* = 3). Left: 3D model synthesized by the algorithm. Green represents the PDOs, and red represents CAR T cells. On the right is the 2D image of CAR T–infiltrating PDOs; CAR T is shown in red with living cell dye. Scale bars: 20 μm. (**E**) Tumor images of the PDOX mouse model treated with CAR T and Moce. (**F** and **G**) Tumor weight and volume of the PDOX mouse model (*n* = 3–5). (**H**) Representative H&E and Ki-67 IHC staining in tumor tissues of the PDOX mouse model established from PC patients’ organoids and treated with CAR T and Moce (*n* = 3). Scale bars: 50 μm. (**I**) Flow cytometry analysis of the proportion of CAR T cells (human CD3^+^CD8^+^) divided into total cells in mouse tumor tissues of PDOX mice after treatment with CAR T and Moce (*n* = 3–5). *P < 0.05, **P < 0.01, ***P < 0.001, by log-rank test (**B**), 1-way ANOVA with Tukey’s multiple-comparison test (**C**, **F**, and **I**) and 2-way ANOVA (**G**). Data are presented as mean ± SD in **C**, **F**, and **I**; mean ± SEM in **G**.

To evaluate the translational potential of ZEB1 in PC chemoimmunotherapy, we selected mocetinostat, an epigenetic inhibitor of ZEB1, to assess its synergistic effect with chemoimmunotherapy in PC. We established an orthotopic allograft mouse model and treated the mice with gemcitabine, gemcitabine+anti–PD-1 (G+P), gemcitabine + mocetinostat (G+M), and gemcitabine + anti–PD-1 + mocetinostat (G+P+M), respectively. We found G+P plus 60 mg/kg mocetinostat treatment (G+P+M) significantly inhibited the tumor growth; however, this regimen didn’t significantly prolong the OS compared with G+M treatment (Supplemental Figure 6, A–C). Since previous clinical trials evaluating the efficacy of gemcitabine in combination with mocetinostat in PC patients did not meet the primary end point due to severe side effects, we decided to explore whether a lower dosage of mocetinostat (30 mg/kg) might improve efficacy. As expected, this treatment strategy significantly reduced tumor volume and improved OS (Figure 6, A and B, and Supplemental Figure 6D), while having markedly less severe side effects, as evidenced by tissue morphology and blood parameters associated with liver and kidney function (Supplemental Figure 6, E and F). To investigate the mechanism by which mocetinostat enhances PC chemoimmunotherapy efficacy, we conducted flow cytometry and IHC to evaluate tumor-infiltrated immune cell profiling. The results showed that the triple-drug treatment led to a dramatic increase in CD8^+^ T cell infiltration (Figure 6C and Supplemental Figure 6G). Meanwhile, levels of neutrophils, often implicated in suppressing anticancer T cell activity across various cancer types, were significantly reduced following G+P+M treatment (Figure 6C and Supplemental Figure 6G). These results indicated that mocetinostat increased chemoimmunotherapy response by remodeling TIME. CAR T cell therapy has shown promising outcomes in hematological malignancies (48). However, the efficacy of CAR T therapy in solid tumors remains limited, particularly in highly desmoplastic PC. To elucidate the impact of mocetinostat on CAR T cell therapy, we constructed patient-derived PC organoids (PDOs) and established CAR T–infiltrated and real-time killing models. High-content confocal laser scanning microscopic images and videos showed that mocetinostat facilitated directional migration and augmented infiltration of CAR T cells into the PDOs within the coculture environment (Figure 6D). Notably, the synergistic effect of mocetinostat and CAR T cells induced dramatic PDO deformation, extensive cell lysis, and cell apoptosis, but the effect was not observed with CAR T cells alone (Supplemental Figure 6, H–L). Given the substantial efficacy of G+P+M in TIME activation, a patient-derived organoid xenograft mouse model (PDOX) was established to investigate whether mocetinostat enhances CAR T cell therapy response in vivo. As shown in Figure 6, E–I, treatment with mocetinostat dramatically improved the antitumor efficacy of CAR T therapy. Thus, mocetinostat reinforced the antitumor immunity and enhanced the efficacy of chemoimmunotherapy and CAR T cell therapy in PC.

### Mocetinostat enhances chemoimmunotherapy sensitivity by targeting HDAC1/2-ZEB1 complex.

Next, we treated resistant cells with mocetinostat and found that it increased the sensitivity of AsPC-R cells to gemcitabine and activated CD8^+^ T cells in vitro (Supplemental Figure 7, A–F). To determine whether the efficacy of mocetinostat in the PC response to chemoimmunotherapy depended on inhibiting the HDAC1/2-ZEB1 functional complex, we performed a co-IP assay. We found that ZEB1 could interact with HDAC1 and HDAC2 (Supplemental Figure 7G). Intriguingly, mocetinostat reduced the stability of ZEB1 and HDAC1 but not HDAC2 (Supplemental Figure 7H). These results indicated that mocetinostat promoted the response to chemoimmunotherapy by disrupting the HDAC1-ZEB1 complex in PC.

### ZEB1 and CXCL16 expression are positively correlated with gemcitabine resistance and associated with poor clinical outcomes.

**Figure 7:**
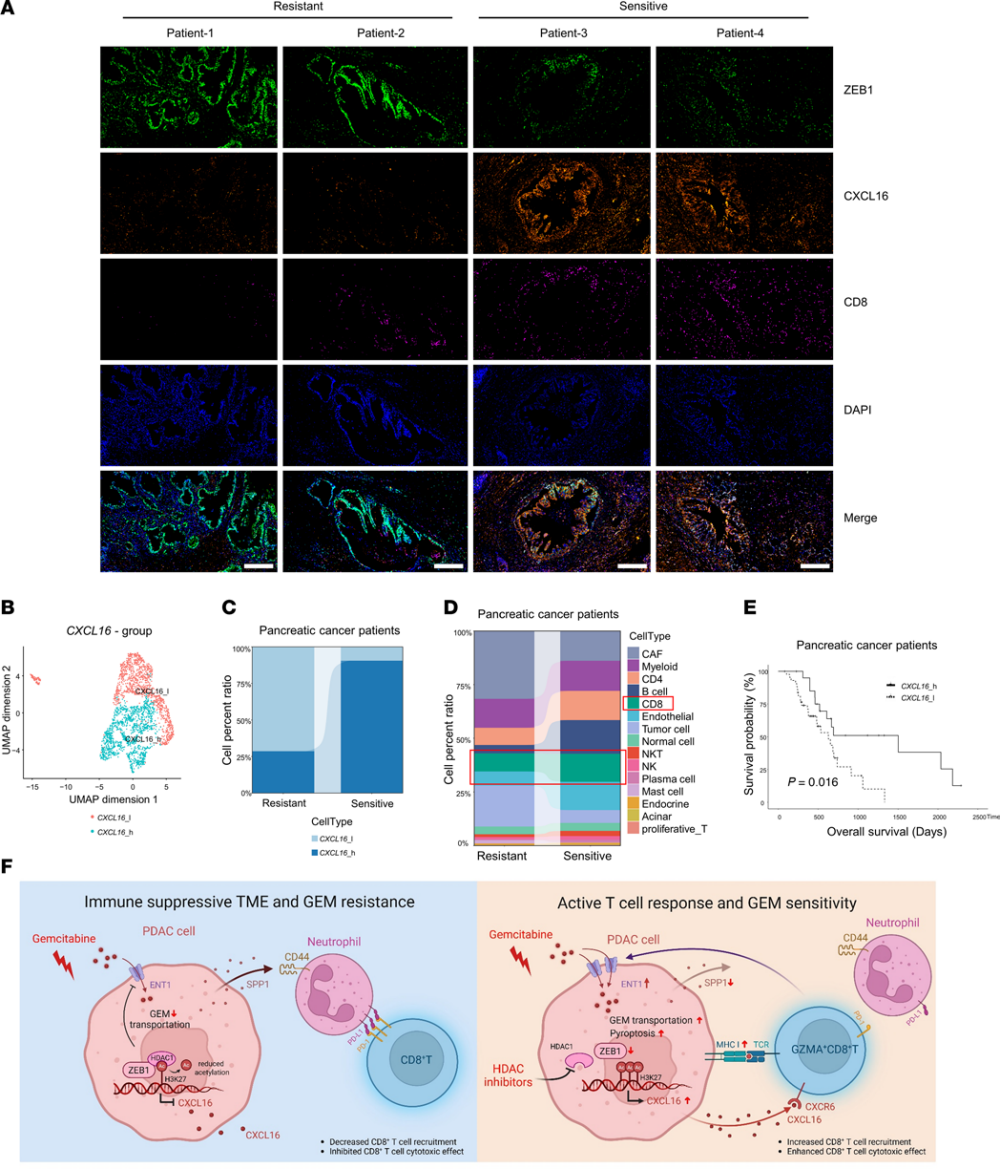
ZEB1 and CXCL16 are associated with chemotherapy resistance, immunosuppression, and prognosis in PC patients. (**A**) Multiple immunofluorescence of ZEB1, CXCL16, and CD8 in tumor tissues of chemosensitive and chemoinsensitive PC patients. Scale bars: 50 μm. (**B**) Based on *CXCL16* expression, tumor cells were categorized into *CXCL16*-high and *CXCL16*-low groups. (**C**) Compared with the chemoresistant group, tumor cells with high *CXCL16* expression were predominantly found in the chemosensitive group. (**D**) Stacked histogram indicates a dramatic increase in the proportion of CD8^+^ T cells in the chemosensitive group. (**E**) In the TCGA dataset, patients receiving adjuvant gemcitabine chemotherapy were stratified into high-*CXCL16*-expression (*n* = 21) and low-expression groups (*n* = 44) based on the optimal cutoff value for *CXCL16* gene expression. Kaplan-Meier survival curves indicate that patients with high *CXCL16* expression exhibited a significantly better prognosis. (**F**) Schematic diagram. Crosstalk between PC cells, CD8^+^ T cells, and neutrophils contributes to tumor immune invasion and gemcitabine resistance through the ZEB1/HDAC1-CXCL16 signaling axis.

We explored the correlation between ZEB1 and CXCL16 expression, as well as CD8^+^ T cell infiltration, and the sensitivity of gemcitabine in patients with PC. ZEB1 expression was positively correlated with gemcitabine resistance, whereas CXCL16 or CD8 expression was negatively correlated (Figure 7A). We further validated these findings using scRNA-Seq of tumor tissues from patients with PC, demonstrating that those with higher CXCL16 expression were more sensitive to gemcitabine treatment (Figure 7, B–E, and Supplemental Figure 7, I and J). Collectively, our data highlight ZEB1 as a central regulator modulating the efficacy of immunotherapy and gemcitabine in PC through its epigenetic regulation of CXCL16 expression and the intratumoral balance of CD8^+^ T cells and neutrophils (Figure 7F).

## Discussion

Chemotherapy inadvertently promotes tumor immune escape, ultimately leading to treatment failure, recurrence, and metastasis. It has been demonstrated that in gemcitabine-resistant PDAC cells, CMTM6 stabilizes PD-L1 expression and inhibits T cell activity (49, 50). In addition, gemcitabine induces the DNA damage response, activating APOBEC3C/3D enzymes, which enhance DNA repair and upregulate the immune checkpoint molecule PD-L1, consequently suppressing T cell function and facilitating immune evasion (51). And EVs secreted by chemotherapy-resistant cells transport miR-21-5p (known to target tumor suppressors such as PDCD4) or PVT1, further inhibiting T cell activity (52).

ZEB1 is one of the key TFs that promote cellular plasticity and tumor metastasis in PC (53, 54). Previous studies showed that ZEB1 induced gemcitabine resistance in PC by activating ITGA3/JNK signaling and downregulating *ENT1* (42). However, the role of ZEB1 in driving chemoimmunotherapy resistance remains elusive. We found that blocking ZEB1 enhanced the efficacy of chemotherapy and immunotherapy (anti–PD-1 therapy and CAR T therapy) by reprogramming the immune microenvironment of PC. Specifically, ZEB1 inhibition increased infiltration of CD8^+^ T cells while decreasing infiltration of neutrophils in vivo. Mechanistically, ZEB1 bound with HDAC1 to regulate the chromatin accessibility of *CXCL16* through histone acetylation, which induced the imbalance of CD8^+^ T cells and neutrophils. Furthermore, our study showed that CD8^+^ T cells reversed chemoresistance by increasing ENT1 expression, echoing the feed-forward loop between chemotherapy and immunotherapy. This work delineates the central role of ZEB1 in reprogramming TIME through epigenetic mechanisms, thereby identifying potential therapeutic targets for enhancing chemotherapy and immunotherapy in PC.

Epigenetic modification, such as acetylation, plays a critical role in driving treatment resistance (55, 56). HDAC1 is a cotranscriptional repressor of ZEB1. However, previous clinical trials evaluating HDAC inhibitors, including mocetinostat — either alone or in combination with chemotherapy or immune checkpoint inhibitors — have failed to demonstrate obvious efficacy in PC and were frequently associated with dose-limiting toxicities, particularly at standard or high doses (57–59). In mouse models, we found that HDAC inhibitor led to a better treatment response when combined with chemotherapy and immunotherapy. We proposed a potential treatment strategy by combining HDAC inhibitor with chemotherapy and immunotherapy, which achieved promising efficacy in PC. Chemokines are critical in regulating immune evasion by facilitating the communication between tumor cells and other cell types in the microenvironment (60–64). We found that CXCL16 recruited and activated CD8^+^ T cells, especially GZMA^+^CD8^+^ T cells, a subpopulation of CD8^+^ T cells that has potent cytotoxicity to tumor cells. GZMA^+^CD8^+^ T cells promote pyroptosis of tumor cells via GSDMD. This is consistent with previous reports showing that cytotoxic lymphocytes can induce pyroptosis in target cells (17). Epigenetic modifications regulate the efficacy of immunotherapy by remodeling TIME (65). We delved into the role of epigenetic modification in regulating CXCL16 expression and delineated that the HDAC1-ZEB1 complex promotes deacetylation of *Cxcl16*, resulting in decreased transcription and expression of CXCL16. This evidence supports the rationale for combining HDAC inhibitors, chemotherapy, and immunotherapy for the treatment of PC.

Furthermore, we noticed decreased infiltration of neutrophils in the tumor microenvironment when HDAC1 or ZEB1 was blocked. Neutrophils induce cancer immune evasion by secreting immune-modulating cytokines, leading to the decreased treatment response to immunotherapy (66). Studies showed that suppressing infiltration of neutrophils and myeloid-derived suppressor cells (MDSCs) by inhibiting CXCR2 resulted in the synergistic antitumor immunity when combined with immunotherapy (61, 67). Intriguingly, senescence-like neutrophils are more potent in driving immunosuppression than their canonical counterparts (68). Moreover, HDAC inhibitor showed a synergistic antitumor effect with CXCR2 inhibitor by eliminating infiltration of senescence-like neutrophils in prostate cancer (68). Future studies may evaluate whether blocking HDAC and neutrophils would increase sensitivity to chemotherapy and immunotherapy in PC.

This study also has limitations. The mechanism by which ZEB1 mediated upregulation of SPP1 remains unclear. Additionally, the potential of other HDAC inhibitors to enhance the efficacy of chemoimmunotherapy in PC warrants further investigation. Currently, no inhibitors are available that specifically target ZEB1. The development of a ZEB1-specific inhibitor would provide a valuable tool to further validate its role in mediating resistance to chemoimmunotherapy.

In conclusion, this study identified a ZEB1-driven reprogramming of the tumor microenvironment that contributed to resistance to both immunotherapy and chemotherapy in PC. While chemotherapy and immunotherapy primarily target tumor cells directly, HDAC inhibitors offer a promising synergistic strategy by modulating key components of TIME. These findings underscore the therapeutic potential of targeting epigenetic modifications, particularly histone acetylation, to overcome treatment resistance and improve outcomes in PC.

## Methods

### Sex as a biological variable.

Our study examined male and female animals, and similar findings are reported for both sexes.

### PDOX mouse model establishment.

PDOs were inoculated onto the back of NSG mice to establish the PDOX F_1_ generation, and subsequently, the F_1_ tumors were chopped into small pieces and inoculated onto the back of nude mice to generate the F_2_ generation. The F_3_ generation was subsequently obtained with the same operation. The PDOX mouse model used in the experiments was the F_3_ generation.

### Construction of CAR T cells.

Construction of EGFR-targeted CAR T cells was performed using a standard protocol. Initial validation of EGFR surface expression was conducted via flow cytometric analysis using APC-conjugated anti-EGFR monoclonal antibody (ABclonal, AB_3662630), demonstrating >90% positivity in both the AsPC-1 cell line and patient-derived organoid models. A second-generation CAR construct was engineered, comprising an anti-EGFR single-chain variable fragment derived from cetuximab; CD8α extracellular hinge and transmembrane domains; 4-1BB (CD137) costimulatory domain; CD3ζ signaling domain; and T2A-linked GFP reporter. The construct was cloned into pLVX-EF1α lentiviral vector and sequence-verified. Lentiviral particles were produced by triple transfection of 293T cells with the CAR transfer vector, psPAX2 packaging plasmid, and pMD2.G envelope plasmid, followed by ultracentrifugation to achieve final titers of 1 × 10^8^ transducing units/mL. For CAR T cell generation, CD8^+^ T lymphocytes were isolated from healthy donor PBMCs via negative selection (EasySep Human CD8^+^ T Cell Isolation Kit, STEMCELL Technologies) and activated with anti-CD3/CD28 Dynabeads at a 1:1 bead-to-cell ratio in the presence of 100 IU/mL recombinant human IL-2; and lentivirus was added to infect the CD8^+^ T cells. One week later, infection efficiency was assessed by measuring the percentage of cells exhibiting GFP using flow cytometry. The positive rate of EGFR–CAR T cells was 50%.

### 10x scRNA sequencing.

According to the user’s manual (CG00315) for the 10x Genomics Chromium Next GEM Single Cell 3′ Kit v3.1 (catalog 1000268), the single-cell suspension was immediately loaded onto a chip to generate GEMs (Gel Bead-in-Emulsion) droplets using the 10x Chromium Controller. Reverse transcription, cDNA amplification, and DNA library construction were performed sequentially according to the protocol. The concentration and fragment size of the libraries were measured using Invitrogen Qubit 4.0 and Agilent 4150 TapeStation. High-throughput sequencing was conducted using high-throughput paired-end 150 bp (PE-150) mode. This work is assisted by OE Biotech Co.

### ATAC-Seq, CUT&Tag-Seq, RNA-Seq, and joint analysis.

ATAC-Seq: AsPC-1 and AsPC GEM cells were collected for preparation of cell suspensions and to obtain cell nuclei. Then, Tn5 transposase was added to cleave DNA into fragments. PCR amplification of DNA fragments and sequencing were performed on the illumina NovaSeq platform. CUT&Tag-Seq: CUT&Tag was performed in AsPC-1 and AsPC-GEM cells by using anti-H3K27ac (Cell Signaling Technology 8173) anti-H3K4me (Cell Signaling Technology 9751) antibodies. The experimental process is as described in Supplemental Methods. Finally, DNA libraries were sequenced on the illumina NovaSeq platform. RNA-Seq: Total RNA was extracted using the Trizol (Invitrogen) method, and RNA purity was detected using NanoDrop One (Thermo Fisher Scientific). Next, mRNA was enriched (T-oligo) and purified, and a library was constructed. Subsequently, sequencing was performed on the MGISEQ-2000 platform. Joint analysis of ATAC-Seq, CUT&Tag-Seq, and RNA-Seq data was performed by Frasergen Bioinformatics Co.

### Human data.

Deidentified clinical information was provided by Peking Union Medical College (PUMC) Hospital.

### Statistics.

Statistical analyses were performed using GraphPad Prism 9 and R 4.1.2. Data from all animal experiments are presented as mean ± SD and analyzed by 1-way ANOVA with Tukey’s multiple-comparison test or unpaired, 2-tailed Student’s *t* test. *P* value less than 0.05 was considered statistically significant. Animal survival analysis was analyzed by log-rank test. Further details about statistical analysis are indicated in Methods and the figure legends. Error bars indicate SEM or SD.

### Study approval.

This study was approved by the IRB at PUMC Hospital. Banked, deidentified tissues were used. Written consent was obtained from all participants. All animal experiments were approved by the IACUC at PUMC Hospital.

### Data availability.

Values for data points in the figures are available in the Supporting Data Values file. scRNA-Seq data generated in this study (GEO GSE281084) and spatial transcriptomics sequencing data (GEO GSE281083) are available in the Gene Expression Omnibus database. FASTQ files of the scRNA-Seq data from human PDACs were obtained from Peng et al. (69) (Genome Sequence Archive [GSA] CRA001160). The data on adjuvant gemcitabine chemotherapy in patients are available in The Cancer Genome Atlas database (TCGA-PAAD, file: “Clinical”). This study did not generate new unique codes. Any additional information required to reanalyze the data reported in this article will be provided upon reasonable request.

## Author contributions

M. Liu and M. Li designed and supervised the study. SZ, YH, ZZ, GL, CZ, YG, FW, YY, HQ, HZ, and WW performed the experiments, analyzed the data and contributed to the visualization of the data. M. Liu and M. Li provided the resources. M. Liu and YH acquired the funding. SZ, YH, and ZZ wrote the original drafts. GL, CZ, YG, FW, YY, HQ, HZ, WW, M. Li, and M. Liu reviewed and edit the manuscript. Co–first authorship was determined by their equal contribution to this study, with SZ listed as the first author because he took the leading role in performing experiments, organizing the figures and writing the original draft.

## Funding support

- National Natural Science Foundation of China, 82273452 and 92474301, to M. Liu.
- National Key Research and Development Program of China, 2023YFC2413200 and 2023YFC2413205, to M. Liu.
- Chinese Academy of Medical Sciences Innovation Fund for Medical Sciences, 2023-I2M-2-004, to M. Liu.
- Non-profit Central Research Institute Fund of Chinese Academy of Medical Sciences, 2022-RC310-01, to M. Liu.
- Beijing Hope Run Special Fund of Cancer Foundation of China, LC2022R05, to M. Liu.
- National Natural Science Foundation of China, 82403706, to YH.
- Fundamental Research Funds for the Central Universities, 3332024047, to YH.
- CPSF Postdoctoral Fellowship Program, grant GZC20240140.

## Supplementary Material

Supplemental data

Unedited blot and gel images

Supporting data values

## Version 1 09/09/2025

In-Press Preview

## Version 2 11/17/2025

Electronic publication
