# Supplementary material for: ZEB1 promotes chemoimmunotherapy resistance in pancreatic cancer models by downregulating chromatin acetylation of CXCL16
Source: J Clin Invest. 2025 Sep 9;135(22):e195970. doi: 10.1172/JCI195970 (PMC12618066; doi:10.1172/JCI195970)
Supplement: Supplemental data [file jci-135-195970-s013.pdf]

Supplementary Materials for

ZEB1 promotes chemo-immune resistance in pancreatic cancer models by downregulating chromatin acetylation of *CXCL16*

Shaobo Zhang[1,†], Yumeng Hu[1,†], Zhijun Zhou[2,3,†], Gaoyuan Lv[1], Chenze Zhang[4], Yuanyuan Guo[1], Fangxia Wang[1], Yuxin Ye[1], Haoran Qi[1], Hui Zhang[5], Wenming Wu[6], Min Li[2,3,*] and Mingyang Liu[1,*]

[1]State Key Laboratory of Molecular Oncology, National Cancer Center/National Clinical Research Center for Cancer/Cancer Hospital, Chinese Academy of Medical Sciences & Peking Union Medical College, Beijing, China

[2]Department of Medicine, the University of Oklahoma Health Sciences Center, Oklahoma City, Oklahoma, 73104, USA

[3]Department of Surgery, the University of Oklahoma Health Sciences Center, Oklahoma City, Oklahoma, 73104, USA

[4]National Key Laboratory of Efficacy and Mechanism on Chinese Medicine for Metabolic Diseases, Beijing Research Institute of Chinese Medicine, Beijing University of Chinese Medicine, Beijing, China

[5]Department of Pathology, Peking Union Medical College Hospital, Chinese Academy of Medical Sciences and Peking Union Medical College, Beijing, China

[6]Department of General Surgery, Peking Union Medical College Hospital, Chinese Academy of Medical Sciences and Peking Union Medical College, Beijing, China

[†]S Zhang, Y Hu and Z Zhou are co–first authors and contributed equally to this work.

***Correspondence to:**

Mingyang Liu, MD, PhD

State Key Laboratory of Molecular Oncology, National Cancer Center/National Clinical Research Center for Cancer/Cancer Hospital, Chinese Academy of Medical Sciences & Peking Union Medical College, Beijing, China

Email: liumy@cicams.ac.cn

Min Li, PhD

Department of Medicine, Department of Surgery

The University of Oklahoma Health Sciences Center

975 NE 10th Street, BRC 1262A, Oklahoma City, OK 73104, USA

Tel: (405) 271-6145, Fax: (405) 271-1476, Email: Min-Li@ouhsc.edu

The PDF file includes:

Supplemental Materials and Methods

Supplemental Figures 1-7

Supplemental Materials and Methods

Cell lines and culture conditions. AsPC-1 cells, MIA-PaCa2 cells were purchased from American Type Culture Collection (ATCC). The KPC cells were developed from $Kras^{G12D}$ $Tp53^{R172H}$ $Pdx\text{-}1\text{-}Cre^{+/+}$ (KPC) mice, and the KPC mice were purchased from Shanghai Model Organisms Center. Gemcitabine resistant cell lines AsPC-GEM and MIA-GEM were constructed by repeatedly stimulating AsPC-1 and MIA-PaCa2 with increasing doses of gemcitabine for a long time.

AsPC-1 cells were cultured in RPMI1640 (HyClone) with 10% FBS (Gibco) and 1% Penicillin-Streptomycin. MIA-PaCa2 cells were cultured in DMEM (Gibco) with 10% FBS (Gibco) and 2.5% HS (Gibco) and 1% Penicillin-Streptomycin. KPC cells were cultured in DMEM (Gibco) with 10% FBS (Gibco) and 1% Penicillin-Streptomycin.

CD8$^+$ T cells migration assay. The CD8$^+$ T cells migration experiment was designed to test the ability of tumor cells to recruit CD8$^+$ T cells. The first co-culture method is to seed AsPC-1 or KPC cells in the 24-well transwell plate, followed by seeding CD8$^+$ T cells to the upper chamber. The other co-culture method is to add AsPC-1 or KPC cells conditioned medium into 24 well plate, followed by seeding CD8$^+$ T cells to the upper chamber. After co-culture for 24h, count the number of CD8$^+$ T cells in the culture medium below.

Co-culture experiments. We performed the co-culture of CD8$^+$ T cells with organoids or tumor cell lines, neutrophils with CD8$^+$ T cells and tumor cell lines. For the co-culture, tumor cells (AsPC-1, KPC) were seeded in the 10 mm dish and adherent growth, and CD8$^+$ T cells and neutrophils were cultured in suspension within the same system. In the end of co-culture, tumor cells, CD8$^+$ T cells and neutrophils were separated by flow cytometry.

In Vitro Tri-Culture System of Tumor Cells, CD8⁺ T Cells and Neutrophils. In vitro tri-culture experiments were performed by isolating neutrophils from mouse bone marrow through density gradient centrifugation, while $CD8^+$ T cells were purified from splenocytes using Mouse $CD8^+$ T Extraction Kit (Stemcell Technologies) according to manufacturer's protocols and activated with anti-CD3/CD28 Dynabeads. For the tri-culture system, tumor cells were seeded in the upper chamber of 0.4 μm transwell inserts, with activated $CD8^+$ T cells and neutrophils co-cultured at a 1:1 ratio in the lower chamber. After 12 hours, tumor cells were removed from the upper chamber, while $CD8^+$ T cells and neutrophils from the lower chamber were separated using $CD8^+$ T cell isolation kit, followed by RNA extraction, cDNA synthesis, and qPCR analysis of cell specific markers (*Gzma*, *Gzmb*, *Gzmk*, *Cd107a*, *Ifng* for T cells; *Icam1*, *Cxcl10*, *Tnfa*, *Cxcr2* for neutrophils).

Stable cell line construction. The *ZEB1* or *CXCL16* knockdown stable cell lines of AsPC-1, MIA-PaCa2 and KPC were developed using lentivirus and selected by adding puromycin (1 μg/ml) or geneticin (400 μg/ml). Three individual lines were selected for each stable cell. KPC-OVA cells were developed using the OVA overexpression lentivirus and selected by adding Blasticidin (2 μg/ml).

Western blot analysis. Collected the cell pellet, added the RIPA lysis buffer (Thermo Fisher Scientific), and incubated on ice for 30 minutes. After Bicinchoninic Acid Assay (Thermo Fisher Scientific), cell lysate protein was prepared and loaded on SDS polyacrylamide gels. Wait until the electrophoresis and membrane transfer processes are completed, membranes were incubated with appropriate primary antibodies at 4°C overnight. After washing with TBST, the membranes were incubated with an HRP secondary antibody for 2 h at room temperature. Finally,

immunoreactive bands were detected using an enhanced chemiluminescent (ECL) plus reagent kit (Thermo Fisher Scientific) in Amersham ImageQuant 800 (Cytiva).

Quantitative real-time PCR. Total RNA was extracted using PureLink RNA Mini Kit (Thermo Fisher Scientific) according to manufacturer's protocols, and 1 μg of RNA was reverse transcribed to cDNA using Evo M-MLV Reverse transcription Kit (Accurate Biology) according to manufacturer's protocols. The mRNA expression was determined using SYBR Select Master Mix (Thermo Fisher Scientific) with ABI QuantStudio 5 Real-Time PCR system. The primer sequences used in qRT-PCR are shown in Supplemental Table 1.

Immunohistochemistry and multiplex immunofluorescence. Human PC tumor tissues and mouse orthotopic allograft tumor tissues were fixed with universal tissue fixative (Servicebio), embedded in paraffin and sectioned. The slides were dried at 70°C for 1h and dewaxed, soaked in 10mM sodium citrate with pH=6, and slightly boiled for 15 min for antigen retrieval. Slides were cooled to room temperature and incubated in the dark with an endogenous peroxide blocker for 15 min, followed by blocking with 10% goat serum for 30 min. Then, slides were incubated with antibody against ZEB1 (Abcam, AB155249), CD8a (Abcam, AB217344; AB199016), CXCL16 (Invitrogen, PA5-115068), Ly6G (Servicebio, GB11229), SPP1 (Servicebio, GB11500), KI67 (Abcam, AB16667) and were incubated overnight at 4°C. The DAB Horseradish Peroxidase Color Development Kit (ZSGB-BIO) was used for tissue color development. For MIF analysis, the section was stained by antibody against ZEB1 (CST, 9963), CXCL16 (Invitrogen, PA5-115068), CD8a (Abcam, AB217344; AB237709), CK19 (Abcam, AB52625). And the staining was conducted using the Multiple fluorescent staining kit (Absin, abs50012) according to manufacturer's protocols.

Immunofluorescence assay. Cells were seeded into 24-well plates with Nune Thermanox coverslip (Thermo Fisher Scientific). After cells grew to 30% - 40% confluency, removed medium, fixed with 4% paraformaldehyde and blocked with 3% bovine serum albumin in PBS. The coverslips were then probed with the ENT1 antibody (ProteinTech, 29862) 4°C overnight, and then incubated with secondary antibody (ProteinTech). Place the coverslip upside down onto a slide with DAPI dye (Thermo Fisher Scientific) for 30 min. The image was captured by cell imaging system EVOS M5000 (Thermo Fisher Scientific).

Immunoprecipitation assay. Use weak IP RIPA lysis buffer (Thermo Fisher Scientific) to lyse cells to obtain protein solution. The supernatant was collected after centrifugation and precleared with IgG and ZEB1 (Abcam, AB155249). The obtained product was eluted using elution buffer and subjected to Western blot analysis. The antibody against HDAC1 and HDAC2 (CST, 34589) for co-immunoprecipitation and the IgG as control (ProteinTech).

Primary human CD8^{+} T cells isolation. As a healthy donor, the first author Shaobo Zhang of this study provided freshly collected venous blood. Next, we first extracted human PBMCs from blood, and then extracted CD8^{+} T cells from PBMCs using Human CD8^{+} T Extraction Kit (Stemcell Technologies) according to manufacturer's protocols. Human CD8^{+} T cells were activated with anti-CD3/CD28 Dynabeads (Thermo Fisher Scientific) at the ratio of 1:1 for 48h and cultured in X-VIVO15 (LONZA) with 5% FBS (Gibco) and 400U/ml IL-2 (MCE) and 1% Penicillin-Streptomycin.

Mouse CD8^{+} T cells and OT1-CD8^{+} T cells isolation. Mouse CD8^{+} T cells were isolated from C57BL/6J mice spleen. We euthanized mice by decapitation and dissected out spleen for grinding and extraction of all cells. Next, we extracted mouse CD8^{+} T cells from all cells using Mouse

CD8$^+$ T Extraction Kit (Stemcell Technologies) according to manufacturer's protocols. The mouse OT1-CD8$^+$ T cells were isolated from OT1-mice spleen in the same way as described above. Mouse CD8$^+$ T cells were activated with anti-Cd3/Cd28 Dynabeads (Thermo Fisher Scientific) at the ratio of 1:1 for 48h, OT1-CD8$^+$ T cells were activated with OVA$_{257\text{-}264}$ (MCE) for 48h. Mouse CD8$^+$ T and OT1-CD8$^+$ T cells were cultured in X-VIVO15 (LONZA) with 5% FBS (Gibco) and 400U/ml IL-2 (MCE) and 1% Penicillin-Streptomycin.

Sample preparation for scRNA sequencing. Freshly harvested orthotopic allograft tumor tissues from mice (KPC-shV, n=1; KPC-shZEB1, n=1) were sectioned into 2–4 mm^3 segments and temporarily stored in tissue preservation solution (MACS, 130-100-008) at 4°C to ensure simultaneous tissue dissociation and sequencing of all samples. The tissues were carefully washed twice with pre-cooled medium (DMEM supplemented with 0.04% BSA) and thoroughly minced into small pieces. Tissue fragments were then digested at 37°C and 22 rpm for approximately 30 min in an enzyme solution containing DMEM, 0.04% BSA, 0.2% collagenase II (Gibco), and 100 μg/L DNase I (Applichem). Additionally, 0.25% trypsin (Gibco) was added for further digestion for 5 min. The resulting cell suspension was filtered through a 40 μm cell strainer, followed by centrifugation at 300 × g for 7 min at 4°C. MACS Red Cell Lysis Solution (130-094-183) was applied to lyse erythrocytes. Cell pellet after washing with medium was resuspended in 200μL PBS with 1% BSA. The Luna Fluorometer (Logos Biosystems) was used to confirmed the number of viable cells, and the cell suspension concentration was then adjusted to 700-1200 cells/μL.

Isolation of mouse neutrophils. 8-12-week-old C57BL/6J mice were sacrificed to obtain femora and tibias. Briefly, the femur and the tibia were removed from all tissue. Then FBS-free RPMI-1640 medium was forced through the cut bone with a syringe to flush out the marrow. Transfer the bone marrow to a clean 15 ml conical tube and centrifuge the bone marrow suspension at 1500

× g for 3 min. Discard the supernatant and resuspend the pellet with FBS-free RPMI-1640 medium. Layer the cell suspension gently on top of a two-layer Percoll (Cytiva) gradient of 65% and 55%. Centrifuge the gradient at 1000 × g for 30 min. Collect the cells from the 55%/65% interface. Wash the cells three times with the FBS-free RPMI-1640 medium. Aseptic techniques were employed throughout the aforementioned steps. The yield was approximately $9 \pm 1 \times 10^6$ cells per mouse.

Luciferase-based cytolysis assay. The AsPC-luciferase and KPC-luciferase cells were seeded in 96-well black opaque plate for 1×10^4 cells (100 μl) every well. After 24h, we added 100 μl CAR-T or OT1-CD8+ T cells at required ratio and co-culture for 24 - 48h. At the end of co-culture, we removed the medium in the well and added 100 μl Steady-Glo luciferase substrate (Promega) and measured the ratio of luminescence intensity of luciferase in PE Victor Nivo Alpha S (Revvity, Inc). The killing level of $CD8^+$ T cells to tumor cells is represented by specific lysis: % killing = 100% × [1 - RLU of well (effector and target cell coculture) / RLU of control well (only target cells)].

CUT&TAG-qPCR. The cleavage under target & tagmentation (CUT&TAG) was performed in AsPC-GEM cells by using the anti-H3K27ac (CST, 8173), anti-HDAC1 (CST, 34589) antibody with the NovoNGS® CUT&Tag 4.0 High-Sensitivity Kit (Novoprotein) following the manufacturer's protocol. After the antibody was pulled down, the target DNA fragment was amplified and determined by qPCR. The sequences for CXCL16 assay are: Forward: 5' ATATGGTGAGGGACAGGAGAGC 3'; Reverse: 5' TGGAGAAGACTACTCAGGGAT 3'.

Dual luciferase assay for promoter activity. The predicted promoter region (from 2,000bp to the transcription start site) of the *CXCL16* gene was ligated to a dual luciferase reporter plasmid pGL4.10. The plasmid PRL-TK was used as an internal reference, and lipo3000 (Thermo Fisher

Scientific) was used for cell transfection. *CXCL16* promoter activity was obtained by measuring the ratio of luminescence intensity of firefly luciferase reporter gene to internal reference plasmid PRL-TK using dual luciferase reporter assay system (Promega).

Flow cytometry. The tumor tissue was dissociated into a single-cell suspension using a tissue dissociation instrument (Miltenyi Biotec) and then washed with PBS containing 1% FBS. Then cells were stained according to antibody's protocols. The stained samples were analyzed using Spectral flow cytometer Cytek NL-CLC3000 (Cytek Biosciences) and data were analyzed using FlowJo software.

scRNA-seq data preprocessing. 10x Genomics generated FASTQ files were processed and aligned to mm10 mouse reference genome using Cell Ranger software (version 7.0.1), with unique molecular identifier (UMI) counts summarized for each barcode. The row UMI count matrix was then analyzed using Seurat (version 5.1.0) R package in R (version 4.3.3). Low-quality cells and potential multiplet captures (defined as cells with <500 detectable genes, <1000 UMIs, >60,000 UMIs, or >15% of transcripts mapping to mitochondrial genes) were excluded. The DoubletFinder package (version 2.0.3) was used to remove inferred doublets. Data normalization and identification of the top 3000 highly variable genes (HVGs) were performed using the SCTransform package. Principal component analysis (PCA) was conducted for dimensionality reduction, with the top 30 PCs used for further analysis. To remove batch effects and achieve well-integrated data across sample origins, the Harmony package (version 1.2.0) was applied with default parameters. Based on batch-corrected Harmony PCs, the data were visualized using the RunUMAP and FindNeighbors functions. Single-cell clusters identified by the FindClusters function, using the Louvain method, were characterized according to known marker genes. Marker genes of subclusters and differentially expressed genes (DEGs) were identified using the

FindAllMarkers and FindMarkers functions. The Nebulosa package (version 1.0.1) was used to generate density plots for visualizing gene expression and gene set scores. The CellChat (version 2.1.1) package was used to estimate and visualize cell-cell communication networks. Pathway enrichment analysis of DEGs was applied by clusterProfiler (version 4.8.3) package.

Construction of PDOs. PDOs were derived from the tumor tissue of patients with PC and cultured in in matrix gel (Corning, 356231) with organoid culture medium (Novoprotein). Fresh surgical specimens were rinsed with DPBS and removed fat and other tissues. The tissues were then cut into small pieces using spring scissors and digested with 1 mg/ml collagenase I (Thermo Fisher Scientific) at 37°C for 1h. The cell suspension was obtained by filtration with a 75 μm filter screen, and the cell precipitation was collected by centrifugation with 500 × g for 5 min. The cells were resuspended with DPBS and centrifuged again under the same conditions. Then, the cells were resuspended with 50 μL of Matrigel in a low-adsorption 24-well plate, fixed at 37°C for 10 minutes, and subsequently supplemented with 500 μL of culture medium.

Orthotopic mouse model. The C57BL/6J, BABL/c, and NSG mice (4-6-week old) used in this study were all purchased from Shanghai Model Organisms Center. We compared the grow of shV, sh*ZEB1*, sh*CXCL16* tumors treated with different drugs. The experimental unit and number were specified in figure legends. Sample size was decided according to the previous studies. Randomisation was used to allocate the mice into experimental units by using a random number generator. To minimize potential confounders, all mice underwent identical surgical procedures. In addition, control and experimental groups were handled in an alternating order to reduce bias related to handling or procedural timing. Group allocation was performed by laboratory personnel who were not involved in the outcome assessment or data analysis, thereby helping to minimize potential bias. The primary outcomes were tumor size and overall survival of the mice. No mice

were excluded in the final analysis. Orthotopic allograft mouse model: KPC cells were digested and resuspended in PBS and mixed with 30% Matrigel (Coning, 354234). After anesthetizing the mice with isoflurane, the skin and peritoneum of the abdomen were incised using high-pressure sterilized surgical instruments. Find and extract the pancreas, inject 5×10^5 KPC cells (30 μl) into the pancreas using a Hamilton syringe, and suture the mouse abdomen. Orthotopic xenograft mouse model: The method is the same as above, and 3×10^6 MIA-PaCa2 cells (50 μl) were injected into the mouse pancreas using a Hamilton syringe. The mice were monitored in a daily base in SPF grade animal laboratories and drug injections began 7 days after the surgery was completed.

Spatial transcriptomic sequencing. Freshly harvested mouse tumor tissues (KPC-shV, n=2; KPC-shZEB1, n=2) were sectioned into appropriately sized fragments, embedded in OCT, and rapidly frozen in liquid nitrogen. Tissue blocks were then subjected to methanol fixation, followed by Hematoxylin & Eosin (H & E) staining, imaging, and destaining, according to the experimental protocol recommended by 10x Genomics (Pleasanton, CA, USA). Subsequently, tissue sections were mounted onto a 10x Visium CytAssist Spatial Gene Expression Slide. This slide contains two capture areas, each measuring 6.5 x 6.5 mm, with approximately 5,000 barcoded spots per area, designed for mRNA capture via oligonucleotides. Following the guidelines from 10x Genomics, probe hybridization and release were performed, and then the probes were transferred to the Visium CytAssist slide. Library construction was subsequently carried out using the Visium CytAssist Spatial Gene Expression for FFPE kit (PN-1000521). The DNA libraries were sequenced using the PE-150 mode. The Space Ranger software (version 2.0.1) from 10x Genomics was used to process FASTQ files and align reads to mm10 mouse reference genome. Tissue-overlaying spots were identified based on the images to distinguish them from background spots. The filtered UMI count matrix was analyzed using Seurat. Applying Sctransform to normalize and

scale data, followed by the identification of the top 3,000 HVGs for subsequent analyses. RCTD (version 1.1.0) was applied to infer cell-type composition of each sport. AddModuleScore was utilized to calculate the expression of selected marker genes for immune cell subsets (T cells: *Ptprc, Cd3d, Cd3e, Cd3g*; Cd8^{+} T cells: *Cd8a*; Gzma effector Cd8^{+} T cells: *Gzma*, *Cxcr6*; Granulocyte: *S100a8, S100a9, Csf3r, Fcgr3, G0s2*), facilitating the identification of immune cell-enriched spots. The R package CellChat was applied to analyze and visualize cell-cell communication network across multiple spatial transcriptomics datasets.

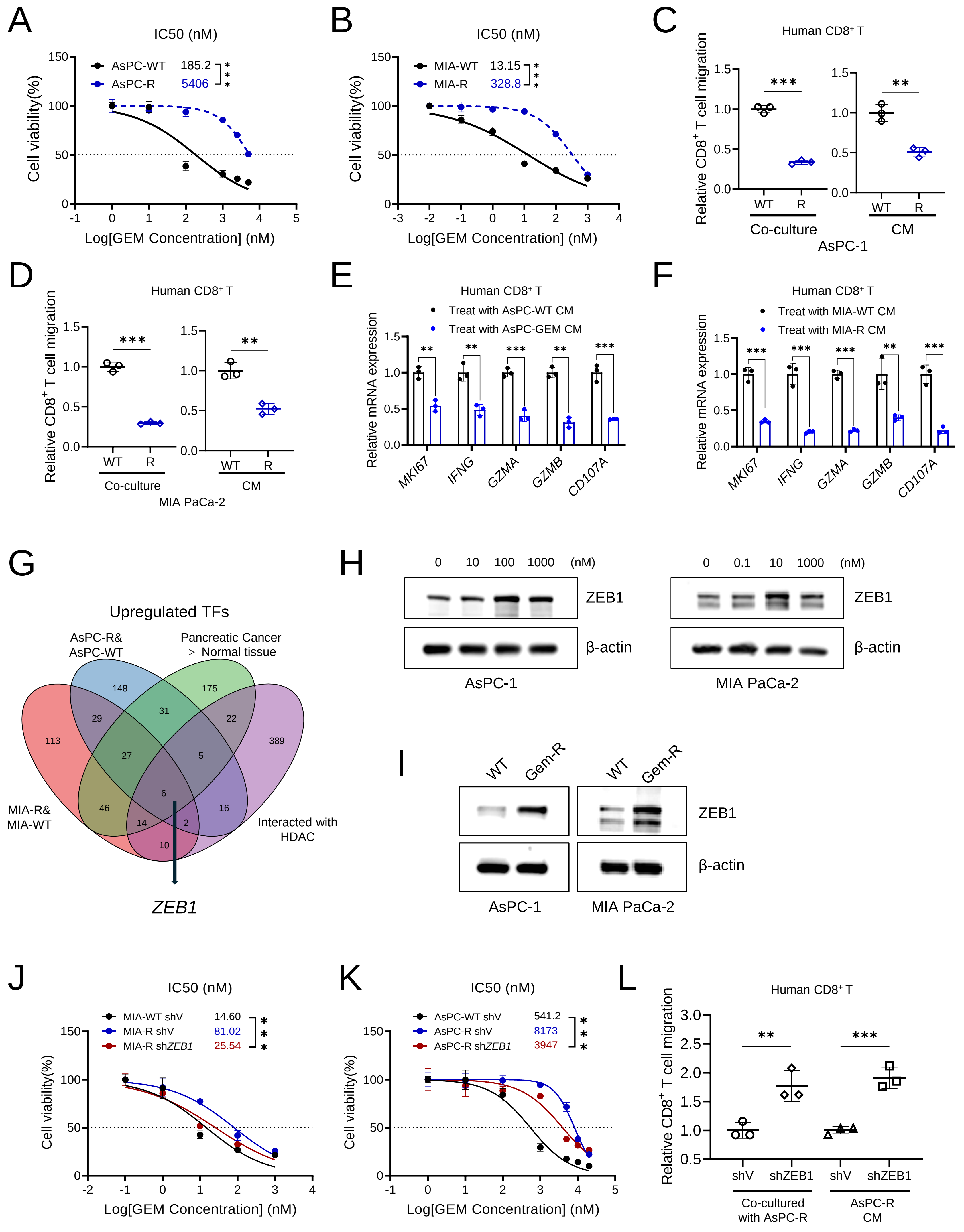

Supplemental Figure 1

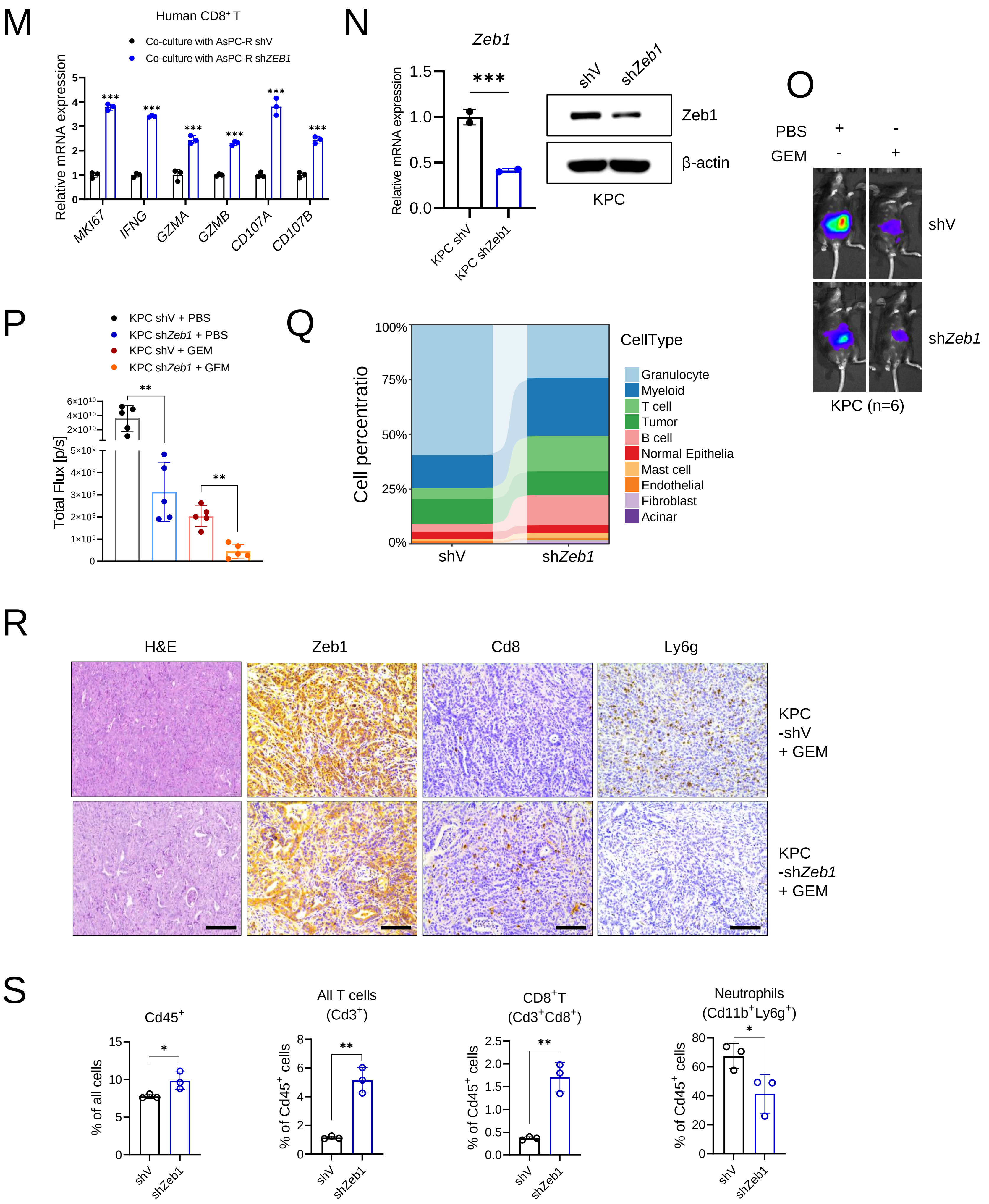

Supplemental Figure 1

Supplemental Figure 1. ZEB1 induced gemcitabine resistance through regulating immune microenvironment of pancreatic cancer.

(A-B) IC50 of WT and chemoresistant cells with treatment of gemcitabine. (C-D) Relative migration of human $CD8^+$ T cells co-incubated with WT and chemoresistant cells. (E-F) Detection of activation markers in human $CD8^+$ T cells after co-culturing with WT and chemoresistant cells. (G) Venn diagrams showing numbers of significantly upregulated TFs overlap with the upregulated genes in tumor tissues, and the genes which can encode proteins that interact with HDACs. *ZEB1* is ranked high. (H) Detection of ZEB1 expression in WT cells treated with gemcitabine. (I) Detection of ZEB1 expression in WT and chemoresistant cells. (J-K) IC50 of chemoresistant cells treated with gemcitabine. (L) Relative migration of human $CD8^+$ T cells co-incubated with AsPC-R (shV or sh*ZEB1*) and treated with the CM. (M) Detection of the activation markers in human $CD8^+$ T cells after co-culturing with AsPC-R (shV or sh*ZEB1*). (N) The knockdown efficiency of *Zeb1* in KPC cells. (O-P) Bioluminescence quantification and statistical analysis of the orthotopic mouse model established from KPC-shV/sh*Zeb1*-luciferase cells (n=5). (Q) Stacked histogram shows the proportion of each cell cluster. (R) H&E and IHC staining of Zeb1, Cd8, and Ly6g in tumor tissues. Scale bar = 50 μm. (S) Flow cytometry analysis of the proportion of $Cd45^+$ cells divided into total cells, and all T cells, $CD8^+$ T cells, neutrophils divided into $Cd45^+$ cells in tumor tissues (n=3). Data are representative of at least 2 or 3 independent experiments. * $P < 0.05$, ** $P < 0.01$, *** $P < 0.001$, by 2-way ANOVA (**A**, **B**, **J** and **K**), unpaired, 2-tailed Student's *t* test (**C**, **D**, **E**, **F**, **L**, **M**, **N** and **S**) and one-way ANOVA with Tukey's multiple comparisons test (**P**). Data represent the mean ± SD.

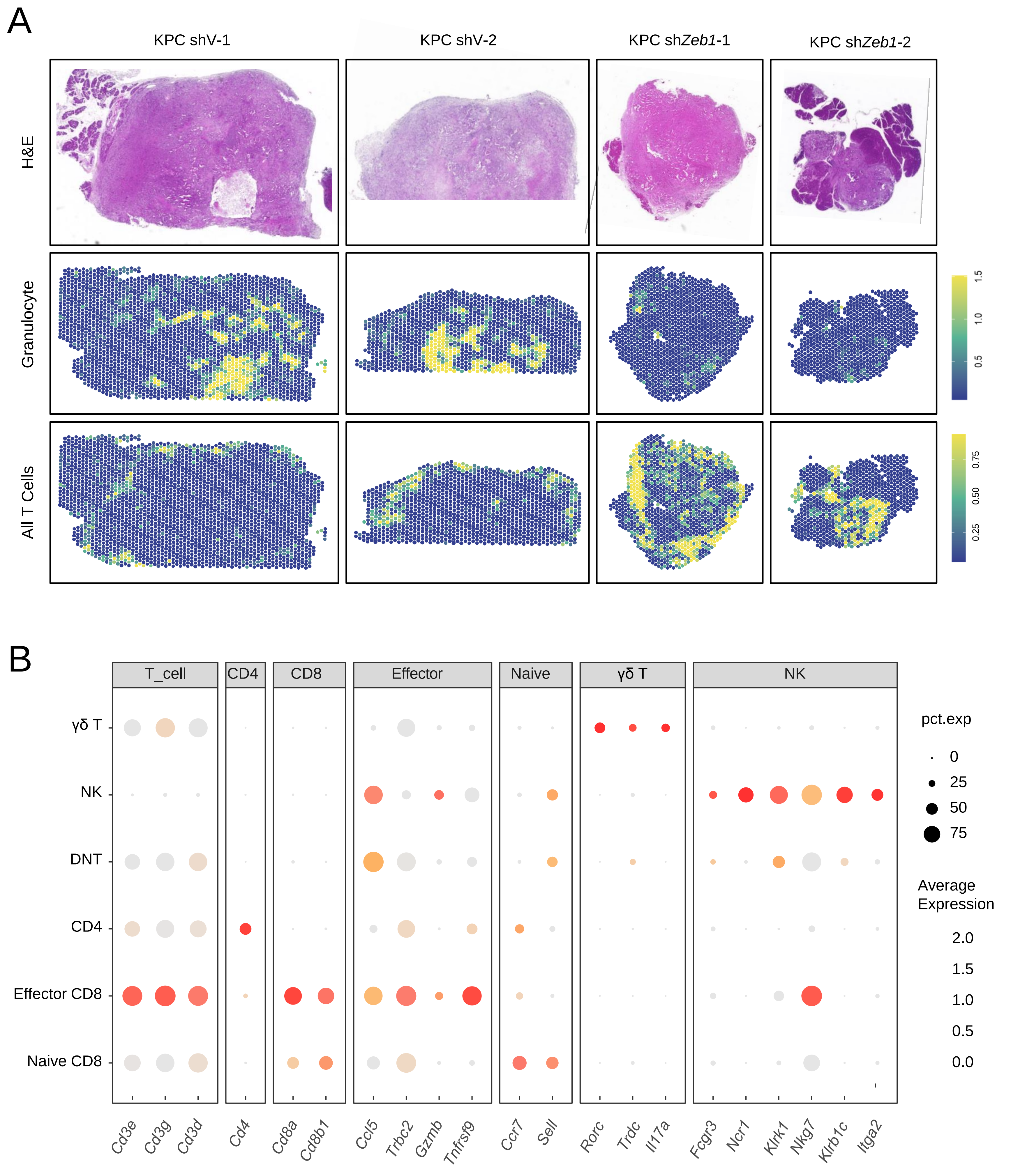

Supplemental Figure 2

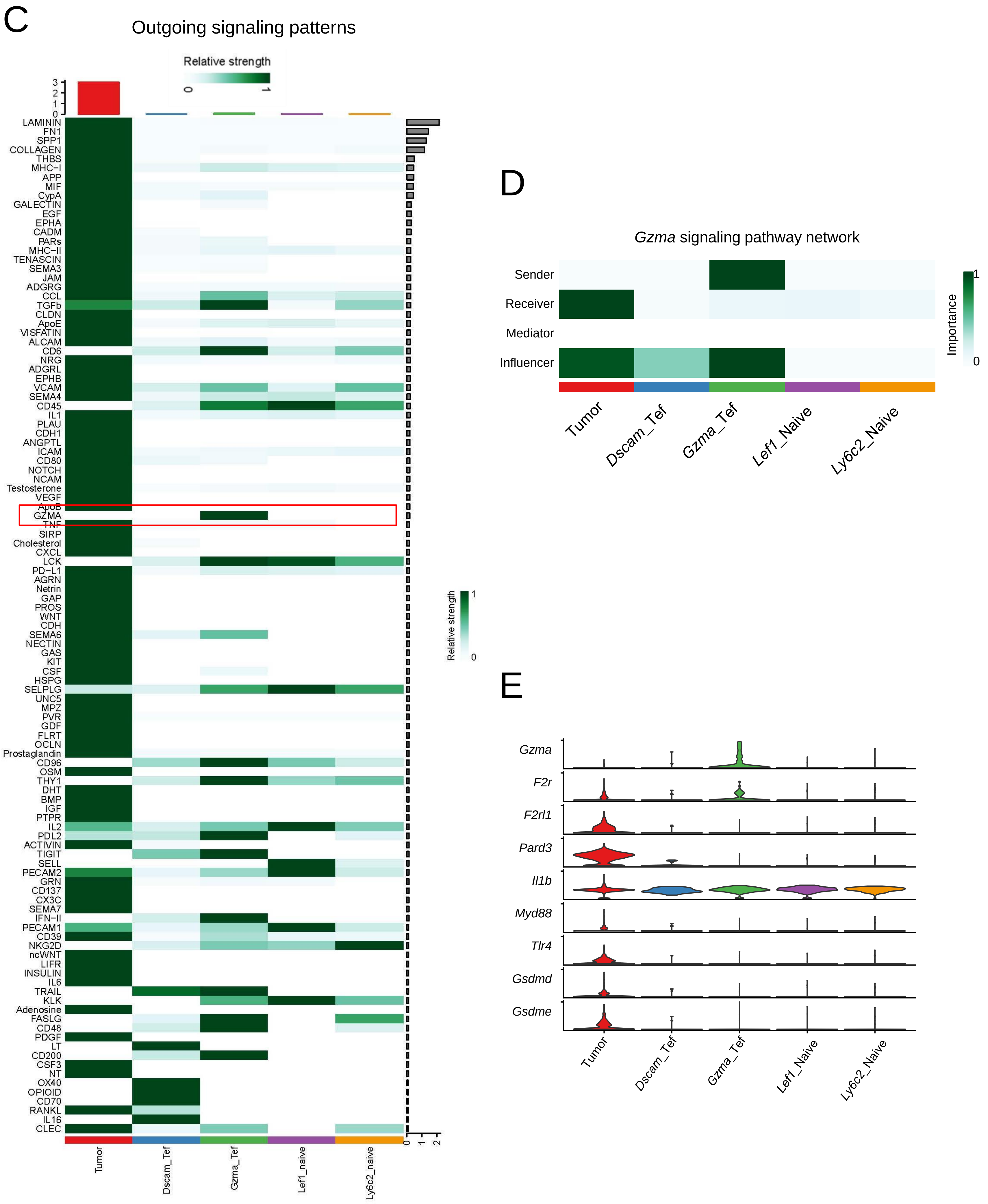

Supplemental Figure 2

Supplemental Figure 2. Gzma$^+$CD8$^+$ T cells are enriched in *Zeb1*-knockdown tumors with gemcitabine treatment.

(A) The first row presents H&E staining of orthotopic allograft mouse models. The second and third rows show the expression scores of selected marker genes for granulocytes and T cells in spatial transcriptomics data, respectively. (B) The dot plot shows representative markers for each T and NK cell subtype. Dot size indicates the proportion of cells within each subtype expressing the specific genes. The intensity of the color corresponds to the relative average expression levels of specific genes. (C) The heatmap shows the strength of outgoing signaling between tumor cells and CD8$^+$ T cell subtypes. Notably, *Gzma* signaling is exclusively secreted by *Gzma* effector T cells. (D) Compared to other CD8$^+$ T cell subtypes, *Gzma* effector T cells are the primary source of *Gzma* signaling, with tumor cells serving as the main recipients. (E) Violin plot shows the gene expression levels of the ligand and receptors involved in *Gzma* signaling.

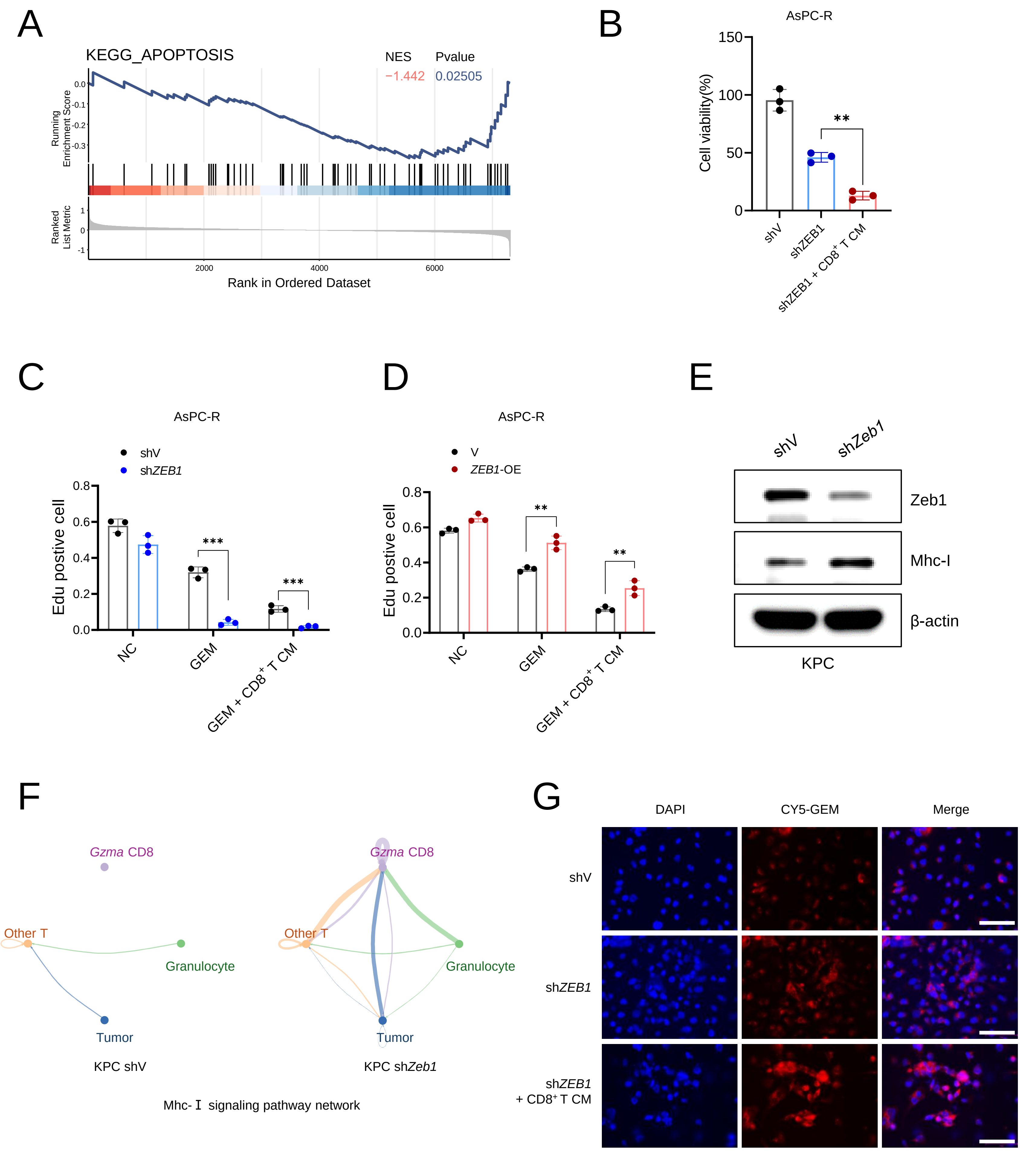

Supplemental Figure 3

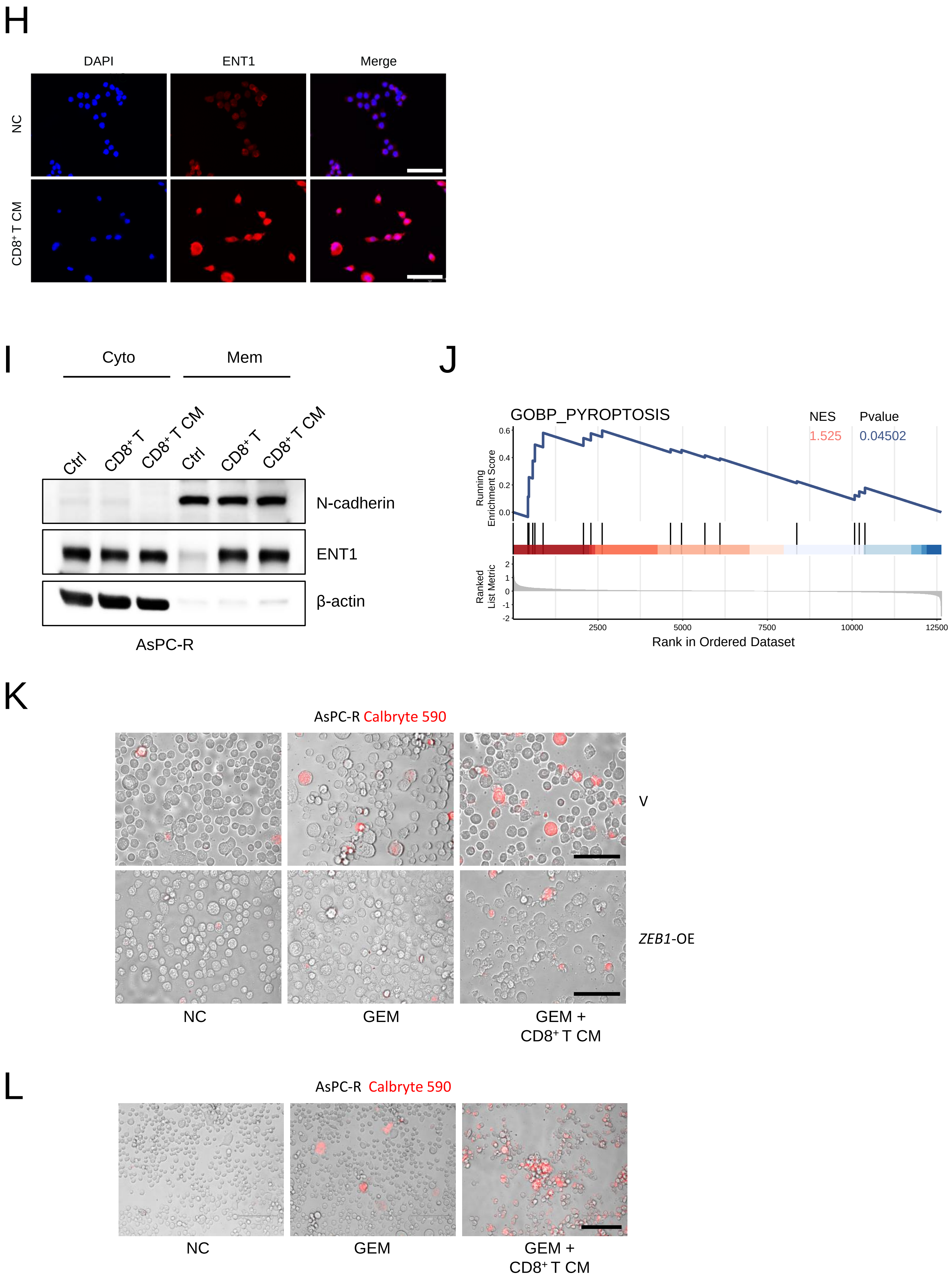

Supplemental Figure 3

Supplemental Figure 3. Blocking of ZEB1 enhances the anti-tumor activity of CD8$^+$ T cells.
(A) Conversely, the apoptosis pathway is downregulated in CD8$^+$ T cells within the KPC-sh*Zeb1* tumor. (B) The cell count assay shows the relative cell viability (%) of AsPC-R cells (shV, sh*ZEB1*) after being treated with a combination of gemcitabine (1000 nM) and CD8$^+$ T CM. (C-D) Detection of EdU-positive cells in AsPC-R cells (shV, sh*ZEB1*, V, *ZEB1*-OE) after being treated with a combination of gemcitabine (1000 nM) and CD8$^+$ T CM. (E) Detection of the expression of Mhc-I in KPC cells (shV, sh*Zeb1*). (F) Circle plots show the communication network of the Mhc-I signaling pathway in KPC-shV and KPC-sh*Zeb1* tumors based on spatial transcriptomics data. (G) Fluorescence imaging reveals the uptake of gemcitabine (red signals) into the AsPC-R cells (shV, sh*Zeb1*) with the combined treatment of Cy5-labeled gemcitabine and CD8$^+$ T CM. Scale bar = 50 µm. (H) Immunofluorescence images showed the expression level of ENT1 in AsPC-R cells treated with CD8$^+$ T CM. Scale bar=50 µm. (I) Detection of ENT1 expression on the cell membrane and in cytoplasm of AsPC-R after treatment with CD8$^+$ T CM. (J) GSEA reveals that the pyroptosis gene set is significantly enriched in the tumor cells of the KPC-sh*Zeb1* tumor. (K) Representative images of AsPC-R cells V, *ZEB1*-OE after being treated with CD8$^+$ T CM or co-cultured with CD8$^+$ T. Scale bar = 25 µm. (L) Representative images of AsPC-1 cells after being treated with CD8$^+$ T CM or co-cultured with CD8$^+$ T. Scale bar=50 µm. Data are representative of at least 2 or 3 independent experiments. * $P < 0.05$, ** $P < 0.01$, *** $P < 0.001$, by 2-way ANOVA (**B**) and unpaired, 2-tailed Student's *t* test (**C**, **D**). Data represent the mean ± SD.

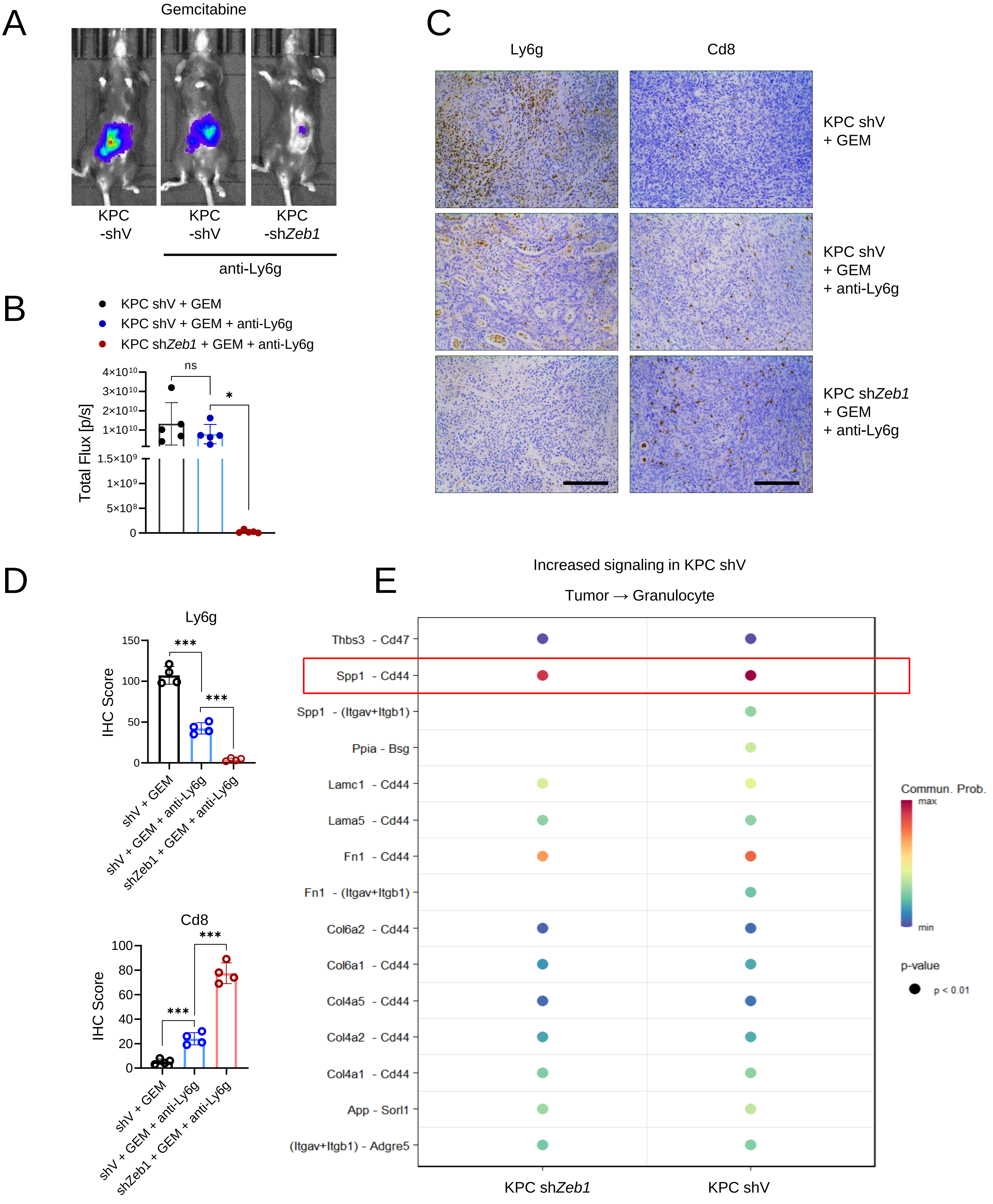

Supplemental Figure 4

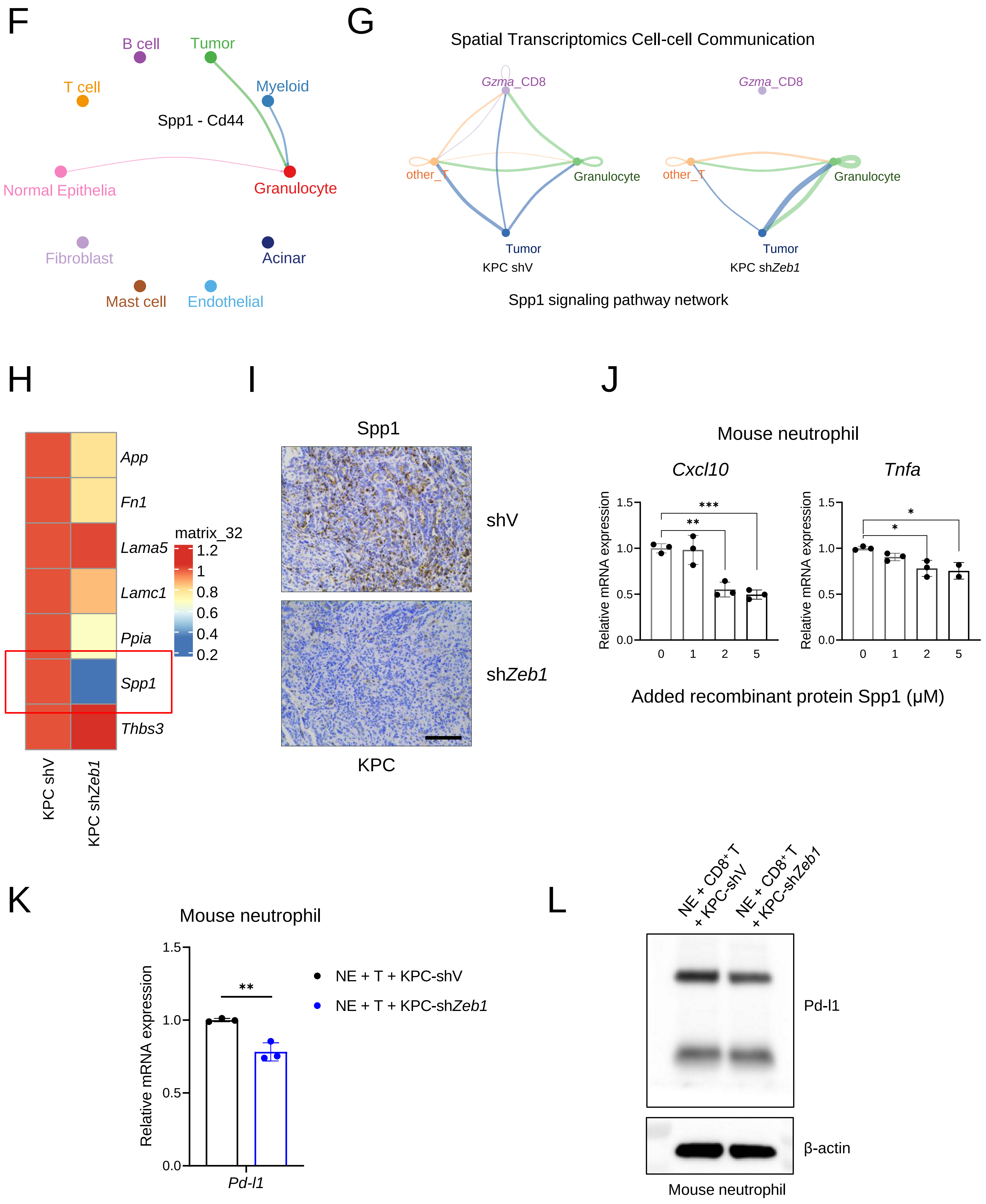

Supplemental Figure 4

Supplemental Figure 4. ZEB1 promotes neutrophil recruitment and drives their polarization toward an immunosuppressive phenotype.

(A-B) Bioluminescence quantification and statistical analysis of the orthotopic allograft mouse model established from KPC-shV/sh*Zeb1*-luciferase cells and treated with gemcitabine and anti-Ly6g (n=5). (C-D) IHC staining of Cd8 and Ly6g in tumor tissues. Scale bar = 50 μm. (E) The dot plot displays all upregulated ligand-receptor (LR) pairs in the KPC shV tumor, with ligands secreted by tumor cells and receptors expressed by granulocytes. (F) The communication network of the Spp1-Cd44 LR pair in the single-cell data from all mice. (G) Circle plots show the communication network of the Spp1 signaling pathway in shV and sh*Zeb1* tumors based on spatial transcriptomics data. (H) The heatmap shows relative expression levels of selected genes. (I) IHC staining of Spp1 in tumor tissues. Scale bar = 50 μm. (J) Detection of the markers of neutrophil after treated with Spp1 recombinant protein for 10 h. (K-L) Detection of the Pd-l1 (Cd274) mRNA and protein expression in mouse neutrophil after co-cultured with mouse $CD8^+$ T cells and KPC-shV or sh*Zeb1* cells. Data are representative of at least 2 or 3 independent experiments. * $P < 0.05$, ** $P < 0.01$, *** $P < 0.001$, by Kruskal-Wallis multiple comparisons test (**B**) and one-way ANOVA with Tukey's multiple comparisons test (**D** and **J**). Data represent the mean ± SD.

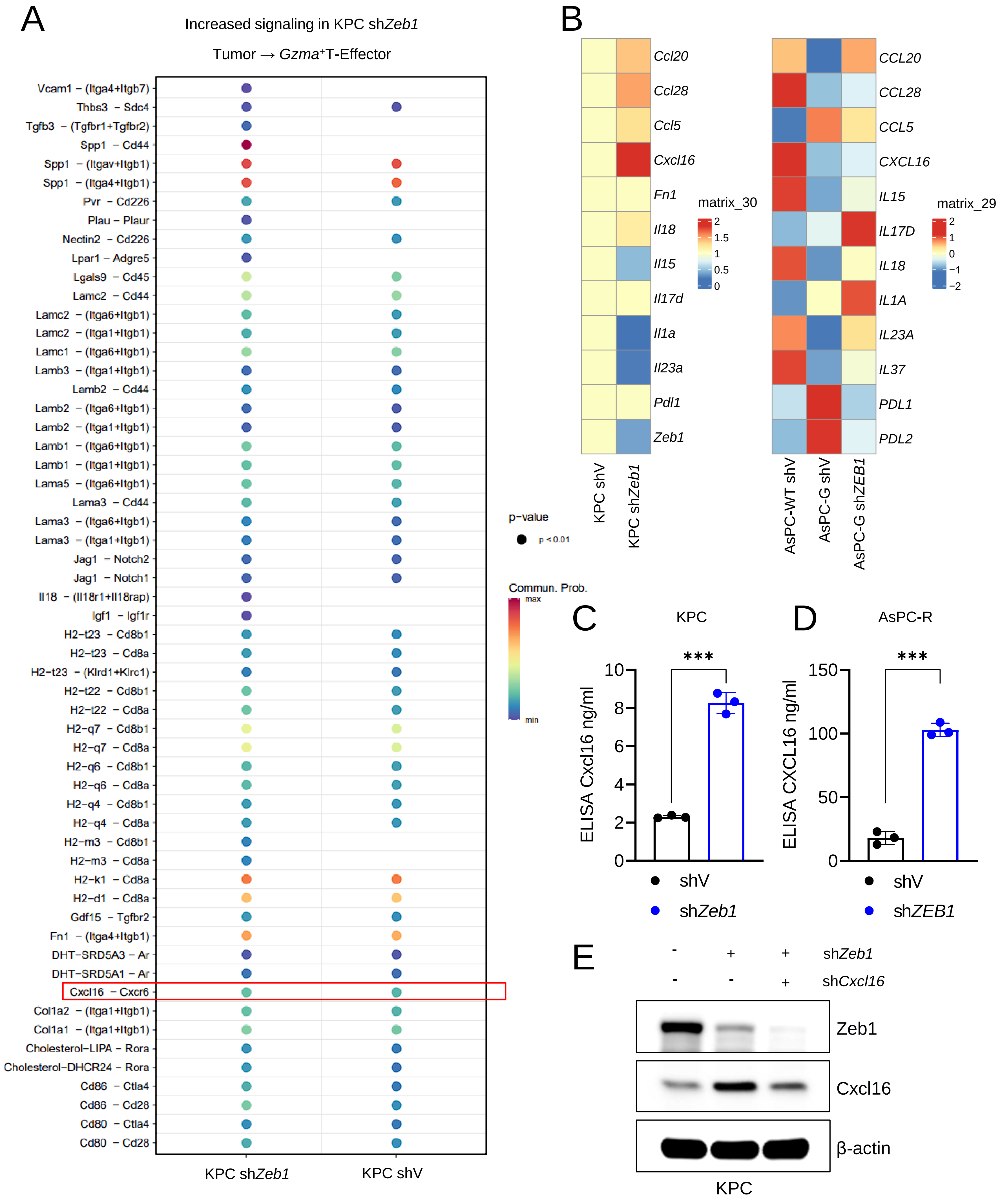

Supplemental Figure 5

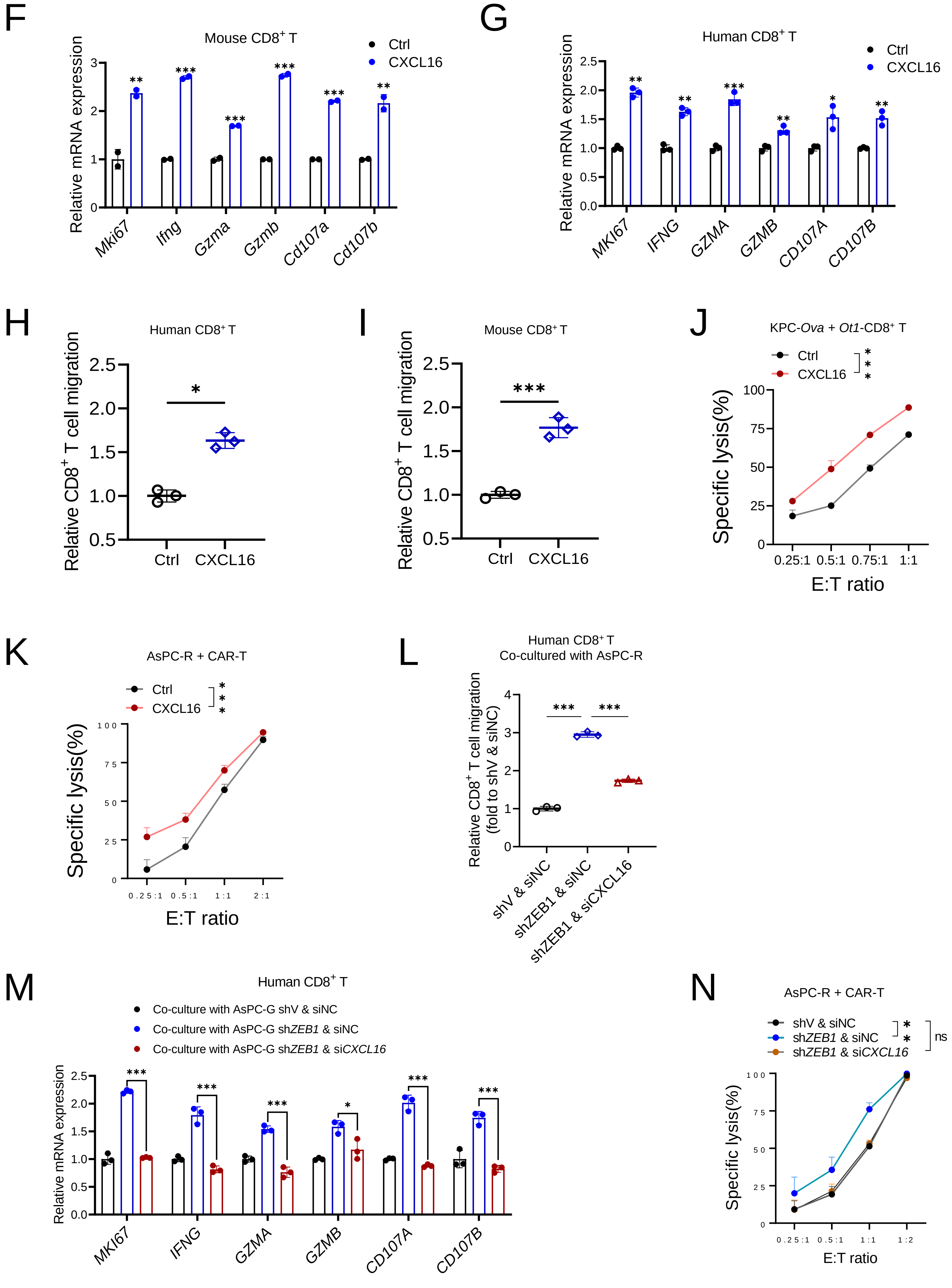

Supplemental Figure 5

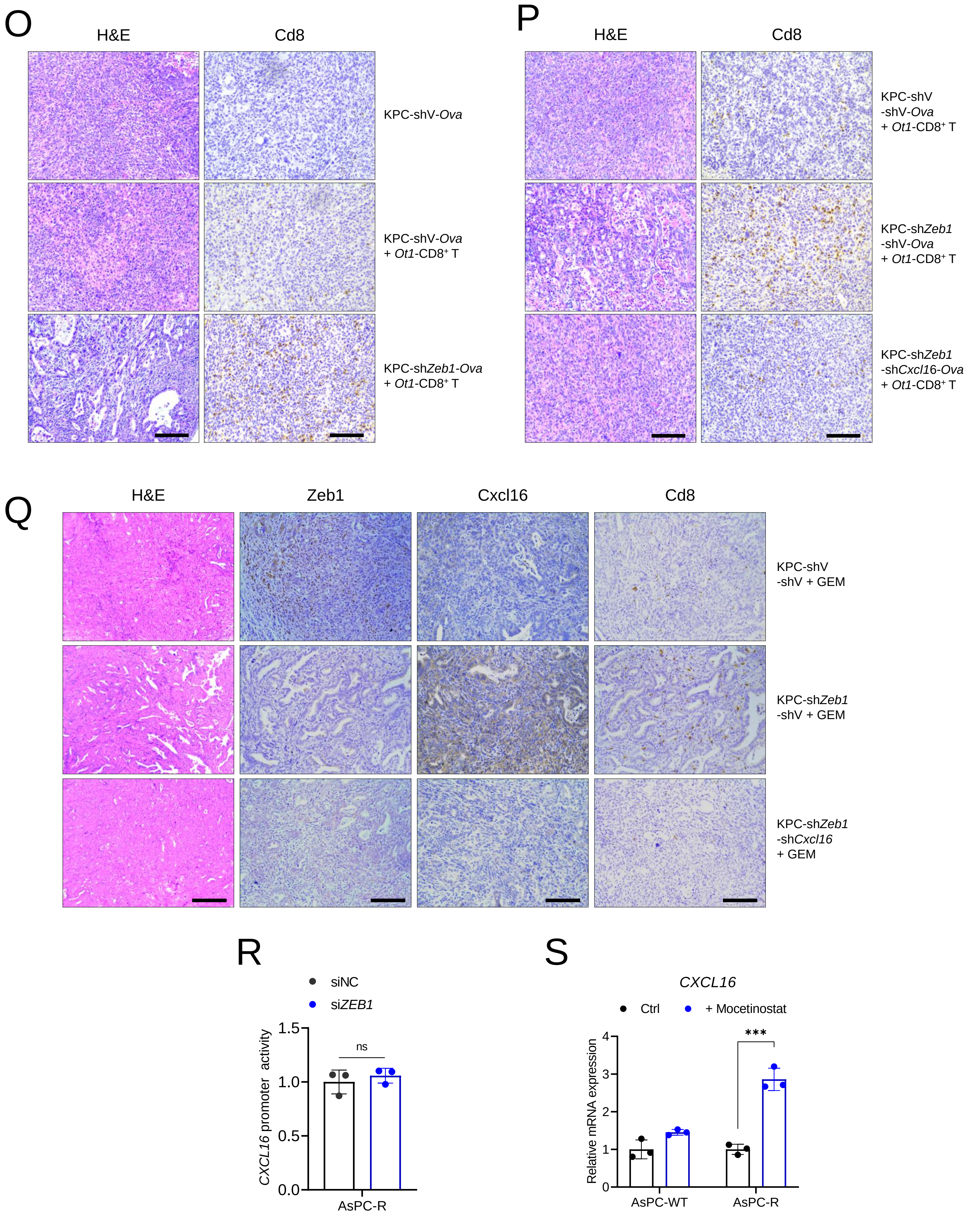

Supplemental Figure 5

Supplemental Figure 5. ZEB1/HDAC1 inhibits the recruitment and function of $CD8^+$ T cells by epigenetically regulating CXCL16.

(A) All increased LR pairs in sh*Zeb1* tumors with secreted by tumor cells and received by *Gzma*$^+$ effector T cells. (B) The heatmap shows relative expression levels of selected genes between shV and sh*zeb1* tumor cells. (C-D) The secretion of CXCL16 in cell culture supernatants was detected by ELISA. (E) Knockdown efficiency of *Cxcl16* in KPC cells. (F-G) Detection of activation markers in $CD8^+$ T cells treated with CXCL16 recombinant protein. (H-I) Relative migration of $CD8^+$ T cells co-incubated with CXCL16 recombinant protein. (J-K) Detection of specific lysis of KPC-*Ova*-luciferase or AsPC-R-luciferase after co-cultured with *Ot1*-$CD8^+$ T or CAR-T added Cxcl16 recombinant protein or not. (L) Relative migration of human $CD8^+$ T cells co-incubated with AsPC-R cells (shV-siNC, sh*ZEB1*-siNC, sh*ZEB1*-si*CXCL16*). (M) Detection activation markers of human $CD8^+$ T cells co-cultured with AsPC-R cells. (N) Detection of specific lysis of AsPC-G-luciferase after co-cultured with CAR-T. (O-P) H&E and IHC staining of Cd8 in tumor tissues. Scale bar=50 μm. (Q) H&E and IHC staining of Zeb1, Cxcl16 and Cd8 in tumor tissues. Scale bar = 50 μm. (R) Dual luciferase reporter assay to examine whether ZEB1 transcriptionally activates CXCL16. (S) Detection of the mRNA level of *CXCL16* in AsPC-WT and AsPC-R treated with Mocetinostat (1μM). Data are representative of at least 3 independent experiments. * $P < 0.05$, ** $P < 0.01$, *** $P < 0.001$, by unpaired, 2-tailed Student's t test (**C**, **F**, **G**, **H**, **I**, **R** and **S**), 2-way ANOVA (**J**, **K** and **N**), and one-way ANOVA with Tukey's multiple comparisons test (**L** and **M**). Data represent the mean ± SD in **C**, **F**, **G**, **H**, **I**, **R**, **S**, **L** and **M**, the mean ± SEM in **J**, **K** and **N**.

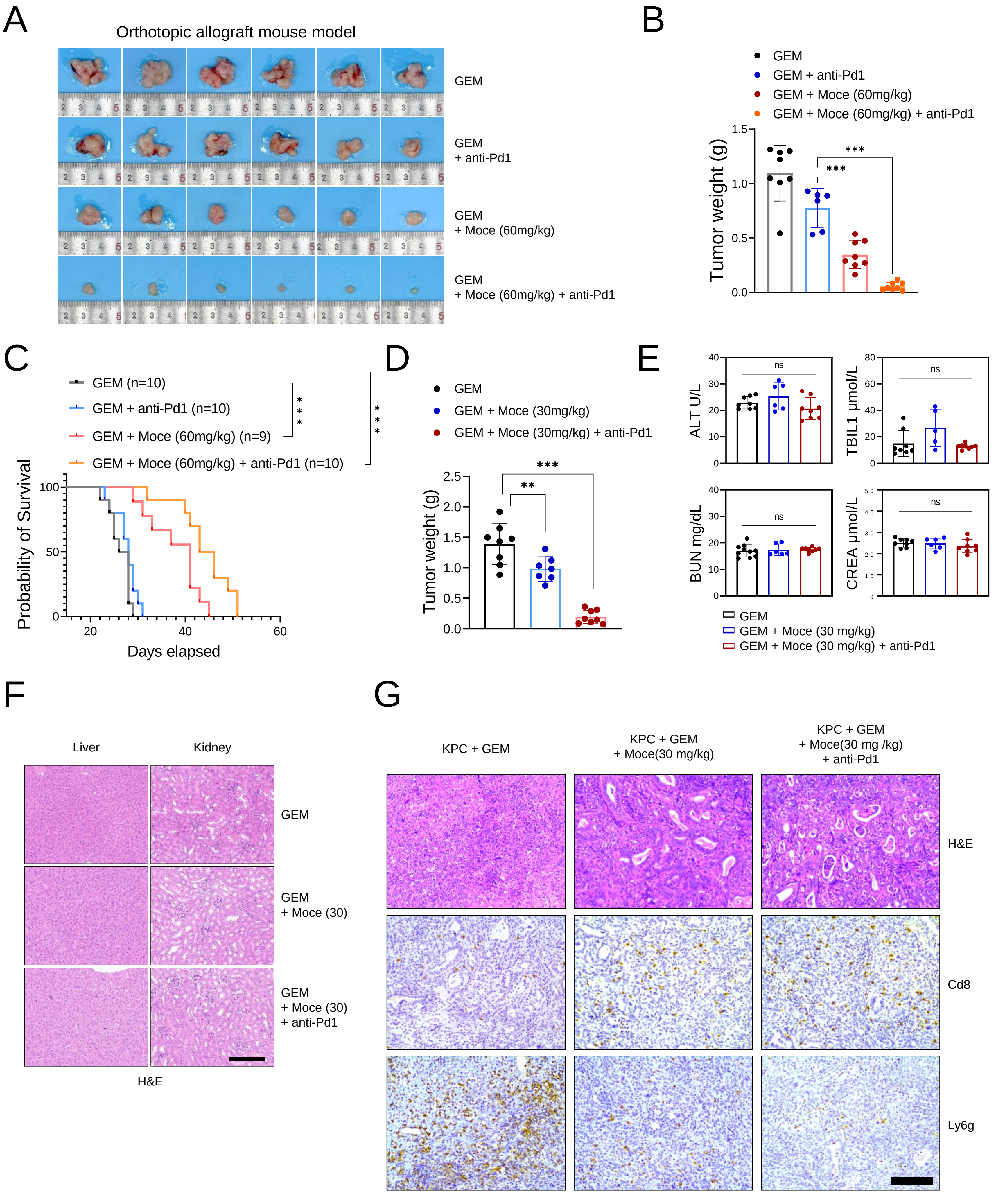

Supplemental Figure 6

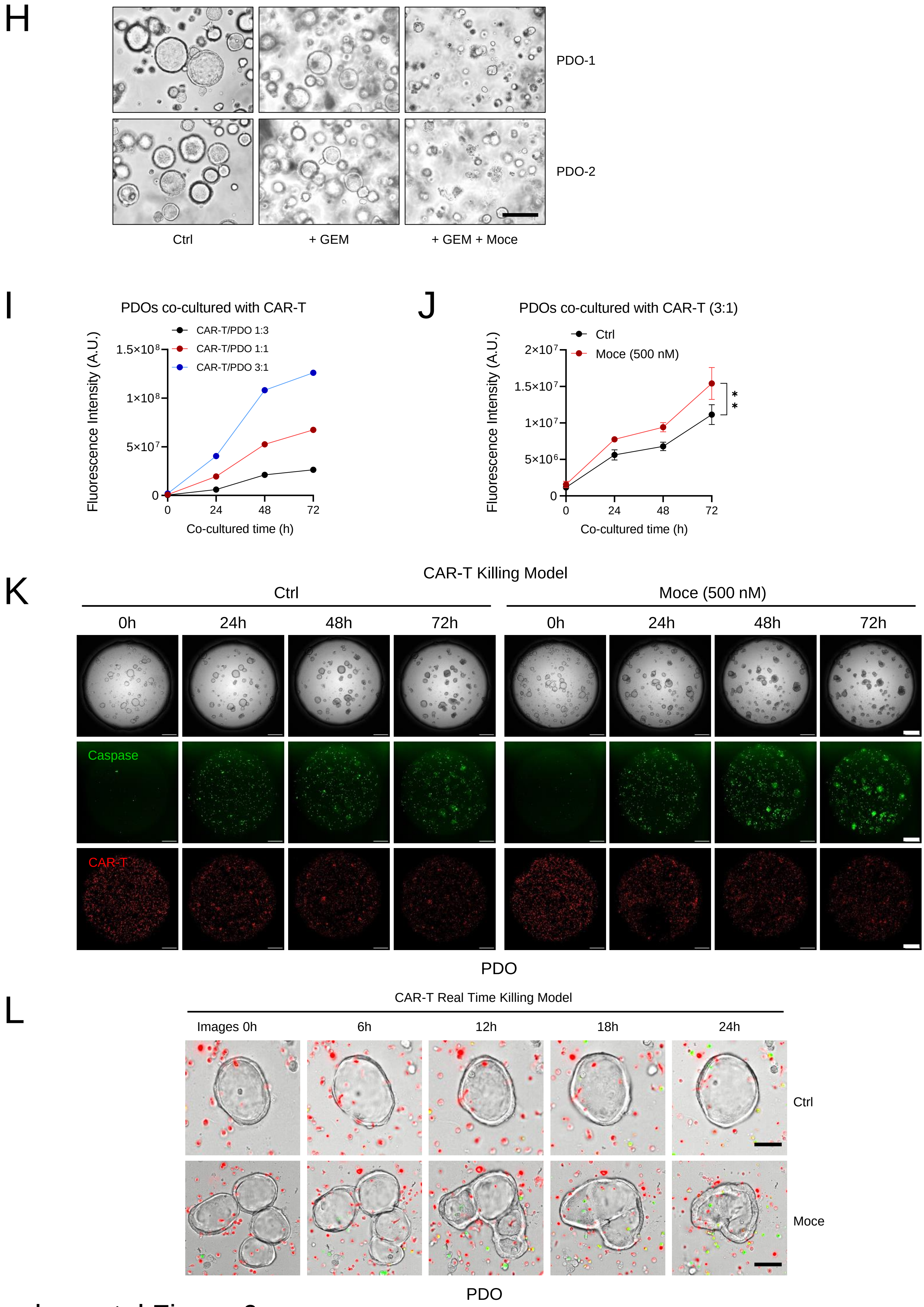

Supplemental Figure 6

Supplemental Figure 6. Mocetinostat enhances the efficacy of chemoimmunotherapy and CAR-T in PC.

(A-B) Representative tumor images and tumor weight of orthotopic allograft mouse model established from KPC cells in each treatment condition (gemcitabine 50 mg/kg, anti-Pd1 10 mg/kg, Moce 60 mg/kg) (n=6-8). (C) Survival of orthotopic allograft mouse model established from KPC cells in each treatment condition (n=9-10). (D) Tumor weight of orthotopic allograft mouse model established from KPC cells in each treatment condition (n=6). (E) Serum from KPC allograft mice treated with gemcitabine and Moce was collected to evaluate the Liver and kidney function (n=6). (F) H&E staining of liver and kidney tissues of KPC allograft mice treated with gemcitabine and Moce (n=6). Scale bar=100 μm. (G) H&E and IHC staining of Cd8 and Ly6g in tumor tissues. Scale bar=50 μm. (H) Representative images of pancreatic cancer organoids with combined treatment of gemcitabine (1000 nM) and Moce (500 nM). Scale bar = 20 μm. (I-K) The model of CAR-T Killing PDOs: CAR-T were used to kill PDOs for 72h after the 24h of Moce (500 nM) treatment of PDOs, calculate the killing ability of CAR-T against PDOs by counting the fluorescence intensity of caspase (Green) and represent CAR-T with red live cell dye. The ratio of CAR-T to PDOs that exhibits the best killing effect in (I) is 3:1. Scale bar=100 μm. (L) The CAR-T Real Time Killing model: CAR-T were used to kill PDOs for 24h after the 24h of Moce (500 nM) treatment of PDOs. Scale bar=20 μm. Data are representative of at least 3 independent experiments. * $P < 0.05$, ** $P < 0.01$, *** $P < 0.001$, by one-way ANOVA with Tukey's multiple comparisons test (**B**, **D** and **E**), 2-way ANOVA (**I** and **J**) and log-rank test (**C**). Data represent the mean ± SD.

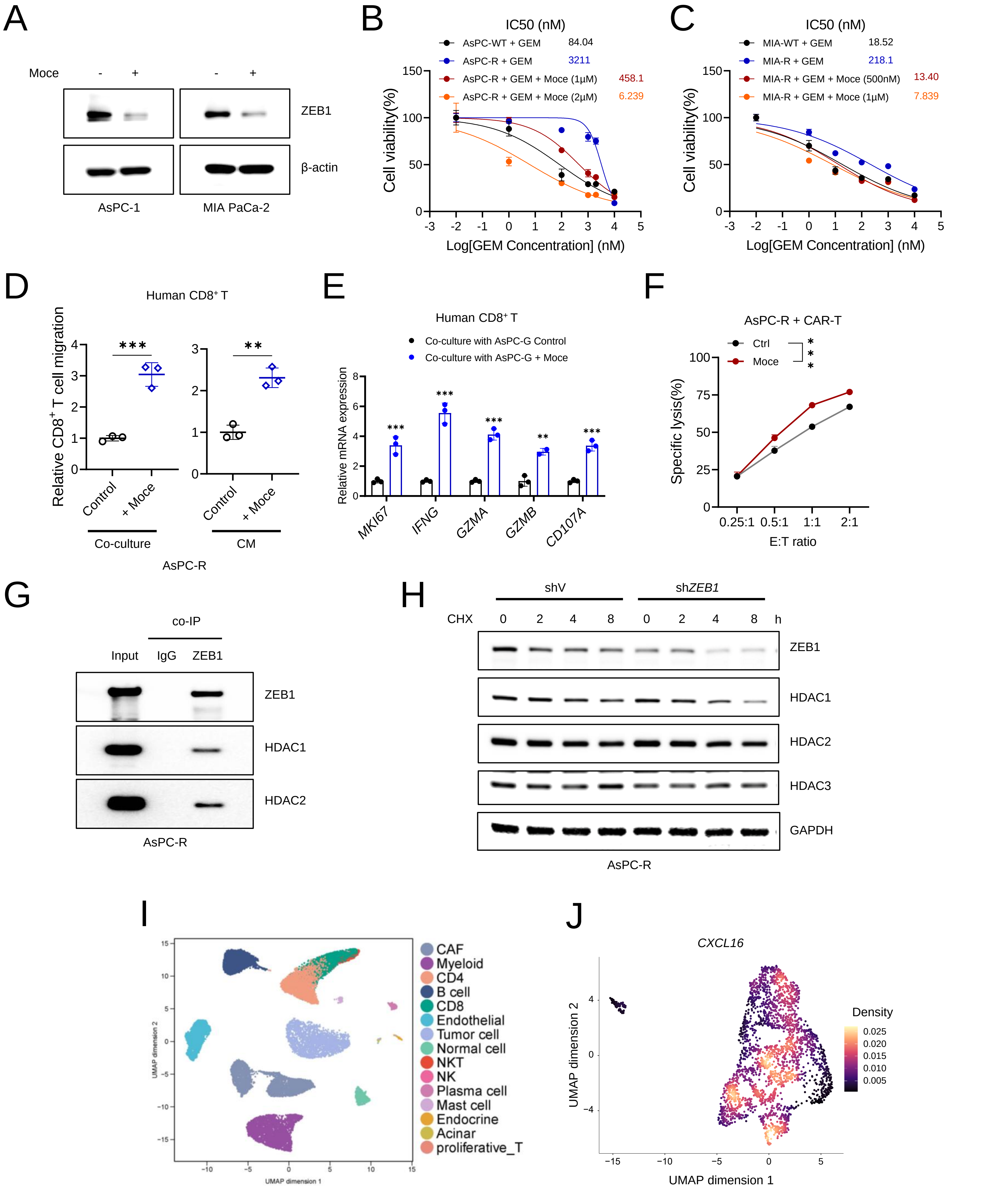

Supplemental Figure 7

Supplemental Figure 7. ZEB1 and CXCL16 are associated with chemotherapy resistance, immunosuppression, and prognosis in PC patients.

(A) Detection of ZEB1 expression in AsPC-1, MIA-PaCa2 cells after treatment with Mocetinostat by Western blot. (B-C) IC50 of AsPC-R and MIA-R with combined treatment of gemcitabine and Mocetinostat for 48h. (D) Relative migration of human $CD8^+$ T cells co-incubated with AsPC-R treated with Mocetinostat (1000 nM). (E) Detection of activation markers in human $CD8^+$ T cells by qPCR after co-cultured with AsPC-R treated with Mocetinostat (1000 nM). (F) Specific lysis of AsPC-R-luciferase treated with Mocetinostat (1000 nM), and co-cultured with CAR-T for 48h. (G) Co-IP assay showed the interaction between ZEB1 with HDAC1 and HDAC2. (H) Detection of ZEB1, HDAC1, HDAC2, HDAC3 protein stability in AsPC-R cells (shV and shZEB1) after treatment with CHX (Cycloheximide, inhibitors of protein synthesis) by Western blot. (I) The UMAP shows fifteen major cell types identified in human PDAC tissues from scRNA-seq data, obtained from three chemo-sensitive and three chemo-resistant patients. (J) Tumor cells were segregated for further analysis. The density plot shows the expression levels of *CXCL16*. Data are representative of at least 2 or 3 independent experiments. * $P < 0.05$, ** $P < 0.01$, *** $P < 0.001$, by unpaired, 2-tailed Student's t test (**D** and **E**), 2-way ANOVA (**B**, **C** and **F**), Data represent the mean ± SD in **B**, **C**, **D** and **E**, the mean ± SEM in **F**.

Supplemental Table 1

qPCR-Primer:	
Primer: human *ZEB1* Forward:	GATGATGAATGCGAGTCAGATGC
Primer: human *ZEB1* Reverse:	ACAGCAGTGTCTTGTTGTTGT
Primer: human *CXCL16* Forward:	CCCGCCATCGGTTCAGTTC
Primer: human *CXCL16* Reverse:	CCCCGAGTAAGCATGTCCAC
Primer: human *GZMA* Forward:	TCTCTCTCAGTTGTCGTTTCTCT
Primer: human *GZMA* Reverse:	GCAGTCAACACCCAGTCTTTTG
Primer: human *GZMB* Forward:	CCCTGGGAAAACACTCACACA
Primer: human *GZMB* Reverse:	GCACAACTCAATGGTACTGTCG
Primer: human *IFNG* Forward:	TCGGTAACTGACTTGAATGTCCA
Primer: human *IFNG* Reverse:	TCGCTTCCCTGTTTTAGCTGC
Primer: human *Ki67* Forward:	GGGCCAATCCTGTCGCTTAAT
Primer: human *Ki67* Reverse:	GTTATGCGCTTGCGAACCT
Primer: human *CD107A* Forward:	GAAAATGCCACTTGCCTTTATGC
Primer: human *CD107A* Reverse:	AGGAAAAGCCAGGTCCGAAC
Primer: human *CD107B* Forward:	GAAAATGCCACTTGCCTTTATGC
Primer: human *CD107B* Reverse:	AGGAAAAGCCAGGTCCGAAC
Primer: human *CCL5* Forward:	CCAGCAGTCGTCTTTGTCAC
Primer: human *CCL5* Reverse:	CTCTGGGTTGGCACACACTT
Primer: human *CCL20* Forward:	TGCTGTACCAAGAGTTTGCTC
Primer: human *CCL20* Reverse:	CGCACACAGACAACTTTTTCTTT
Primer: human *CCL28* Forward:	TGCACGGAGGTTTCACATCAT
Primer: human *CCL28* Reverse:	TTGGCAGCTTGCACTTTCATC

Primer: human *PDL1* Forward:	TGGCATTTGCTGAACGCATTT
Primer: human *PDL1* Reverse:	TGCAGCCAGGTCTAATTGTTTT
Primer: human *PDL2* Forward:	ATTGCAGCTTCACCAGATAGC
Primer: human *PDL2* Reverse:	AAAGTTGCATTCCAGGGTCAC
Primer: human *IL15* Forward:	CGCAGACATCTGGGTCAAGAG
Primer: human *IL15* Reverse:	TTGTTCAACACGCACTCCGT
Primer: human *IL17D* Forward:	GCCCTGGGCCTACAGAATC
Primer: human *IL17D* Reverse:	CGCCCTGTTTGTCGATGCT
Primer: human *IL18* Forward:	TCTTCATTGACCAAGGAAATCGG
Primer: human *IL18* Reverse:	TCCGGGGTGCATTATCTCTAC
Primer: human *IL1A* Forward:	TGGTAGTAGCAACCAACGGGA
Primer: human *IL1A* Reverse:	ACTTTGATTGAGGGCGTCATTC
Primer: human IL23A Forward:	CTCAGGGACAACAGTCAGTTC
Primer: human *IL23A* Reverse:	ACAGGGCTATCAGGGAGCA
Primer: human *IL37* Forward:	TTCTTTGCATTAGCCTCATCCTT
Primer: human *IL37* Reverse:	CGTGCTGATTCCTTTTGGGC
Primer: human *GAPDH* Forward:	TGTGGGCATCAATGGATTTGG
Primer: human *GAPDH* Reverse:	ACACCATGTATTCCGGGTCAAT
Primer: mouse *Zeb1* Forward:	ACCGCCGTCATTTATCCTGAG
Primer: mouse *Zeb1* Reverse:	CATCTGGTGTTCCGTTTTCATCA
Primer: mouse *Ki67* Forward:	ATCATTGACCGCTCCTTTAGGT
Primer: mouse *Ki67* Reverse:	GCTCGCCTTGATGGTTCCT
Primer: mouse *Ifng* Forward:	GCCACGGCACAGTCATTGA
Primer: mouse *Ifng* Reverse:	TGCTGATGGCCTGATTGTCTT

Primer: mouse *Gzma* Forward:	GGGGCTCACTCAATCAATAAGG
Primer: mouse *Gzma* Reverse:	CATCCTGCTACTCGGCATCT
Primer: mouse *Gzmb* Forward:	TCTCGACCCTACATGGCCTTA
Primer: mouse *Gzmb* Reverse:	TCCTGTTCTTTGATGTTGTGGG
Primer: mouse *Gzmk* Forward:	TGGCTGGCGTTTATATGTCTTC
Primer: mouse *Gzmk* Reverse:	TCTGGGAAACCAAGAGTAGCA
Primer: mouse *Cd107a* Forward:	CAGCACTCTTTGAGGTGAAAAAC
Primer: mouse *Cd107a* Reverse:	CCATTCGCAGTCTCGTAGGTG
Primer: mouse *Cd107b* Forward:	TGTATTTGGCTAATGGCTCAGC
Primer: mouse *Cd107b* Reverse:	TATGGGCACAAGGAAGTTGTC
Primer: mouse *Icam1* Forward:	GTGATGCTCAGGTATCCATCCA
Primer: mouse *Icam1* Reverse:	CACAGTTCTCAAAGCACAGCG
Primer: mouse *Cxcl10* Forward:	CCAAGTGCTGCCGTCATTTTC
Primer: mouse *Cxcl10* Reverse:	GGCTCGCAGGGATGATTTCAA
Primer: mouse *Cxcr2* Forward:	ATGCCCTCTATTCTGCCAGAT
Primer: mouse *Cxcr2* Reverse:	GTGCTCCGGTTGTATAAGATGAC
Primer: mouse *Tnfa* Forward:	CAGGCGGTGCCTATGTCTC
Primer: mouse *Tnfa* Reverse:	CGATCACCCCGAAGTTCAGTAG
Primer: mouse *Cxcl16* Forward:	CCTTGTCTCTTGCGTTCTTCC
Primer: mouse *Cxcl16* Reverse:	TCCAAAGTACCCTGCGGTATC
Primer: mouse *Spp1* Forward:	ATCTCACCATTCGGATGAGTCT
Primer: mouse *Spp1* Reverse:	TGTAGGGACGATTGGAGTGAAA
Primer: mouse *Pdl1* Forward:	GCTCCAAAGGACTTGTACGTG
Primer: mouse *Pdl1* Reverse:	TGATCTGAAGGGCAGCATTTC

Primer: mouse *Ccl5* Forward:	GCTGCTTTGCCTACCTCTCC
Primer: mouse *Ccl5* Reverse:	TCGAGTGACAAACACGACTGC
Primer: mouse *Ccl20* Forward:	GCCTCTCGTACATACAGACGC
Primer: mouse *Ccl20* Reverse:	CCAGTTCTGCTTTGGATCAGC
Primer: mouse *Ccl28* Forward:	AGAGTGAGTTCATGCAGCATC
Primer: mouse *Ccl28* Reverse:	CTGCTTCAAAGTACGATTGTGC
Primer: mouse *Fn1* Forward:	ATGTGGACCCCTCCTGATAGT
Primer: mouse *Fn1* Reverse:	GCCCAGTGATTTCAGCAAAGG
Primer: mouse *Il18* Forward:	GACTCTTGCGTCAACTTCAAGG
Primer: mouse *Il18* Reverse:	CAGGCTGTCTTTTGTCAACGA
Primer: mouse *Il15* Forward:	CATCCATCTCGTGCTACTTGTG
Primer: mouse *Il15* Reverse:	GCCTCTGTTTTAGGGAGACCT
Primer: mouse *Il17d* Forward:	AGCACACCCGTCTTCTCTC
Primer: mouse *Il17d* Reverse:	GCTGGAGTTCGCACTGTCC
Primer: mouse *Il1a* Forward:	TCTATGATGCAAGCTATGGCTCA
Primer: mouse *Il1a* Reverse:	CGGCTCTCCTTGAAGGTGA
Primer: mouse *Il23a* Forward:	CAGCAGCTCTCTCGGAATCTC
Primer: mouse *Il23a* Reverse:	TGGATACGGGGCACATTATTTTT
Primer: mouse *App* Forward:	TCCGAGAGGTGTGCTCTGAA
Primer: mouse *App* Reverse:	CCACATCCGCCGTAAAAGAATG
Primer: mouse *Lama5* Forward:	CTGGCGGAGATCCCAATCAG
Primer: mouse *Lama5* Reverse:	GTGTGACGTTGACCTCATTGT
Primer: mouse *Lamc1* Forward:	TGCCGGAGTTTGTTAATGCC
Primer: mouse *Lamc1* Reverse:	TGGTTGTTGTAGTCGGTCAGG

Primer: mouse *Ppia* Forward:	GAGCTGTTTGCAGACAAAGTTC
Primer: mouse *Ppia* Reverse:	CCCTGGCACATGAATCCTGG
Primer: mouse *Thbs3* Forward:	AAGCCGGAACTTTGGGGTG
Primer: mouse *Thbs3* Reverse:	AGTGAGTAAAGCTGTCCGAATCT
Primer: mouse *Gapdh* Forward:	TGGATTTGGACGCATTGGTC
Primer: mouse *Gapdh* Reverse:	TTTGCACTGGTACGTGTTGAT
**shRNA**	
Human shZEB1	CCTCTCTGAAAGAACACATTA
Mouse shZEB1	ATAGAGGCTACAAGCGCTTTA
Mouse shCXCL16	GAGGCAAATGAGAAACAGCAA
**CUT&TAG-qPCR-Primer:**	
CXCL16-F:	ATATGGTGAGGGACAGGAGAGC
CXCL16-R:	TGGAGAAGACTACTCAGGGAT
