# Supplementary material for: ZEB1 promotes chemoimmunotherapy resistance in pancreatic cancer models by downregulating chromatin acetylation of CXCL16
Source: J Clin Invest. 2025 Sep 9;135(22):e195970. doi: 10.1172/JCI195970 (PMC12618066; doi:10.1172/JCI195970)
Supplement: Unedited blot and gel images [file jci-135-195970-s014.pdf]

Figure 3D.

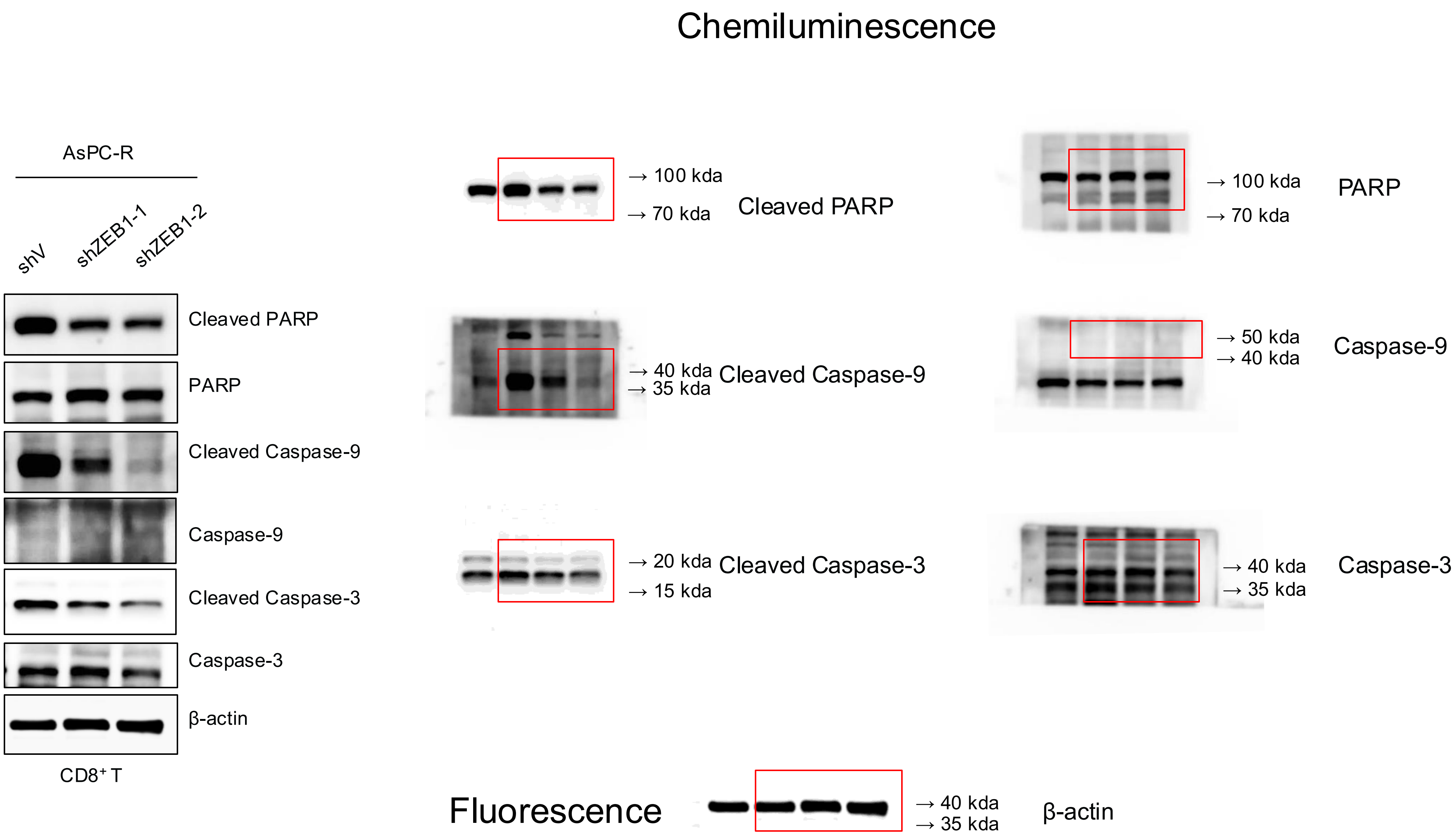
Chemiluminescence
AsPC-R
shV
shZEB1-1
shZEB1-2
Cleaved PARP
PARP
Cleaved Caspase-9
Caspase-9
Cleaved Caspase-3
Caspase-3
β-actin
CD8⁺ T
→ 100 kda
→ 70 kda
Cleaved PARP
→ 100 kda
→ 70 kda
PARP
→ 40 kda
→ 35 kda
Cleaved Caspase-9
→ 50 kda
→ 40 kda
Caspase-9
→ 20 kda
→ 15 kda
Cleaved Caspase-3
→ 40 kda
→ 35 kda
Caspase-3
Fluorescence
→ 40 kda
→ 35 kda
β-actin

Figure 3I.

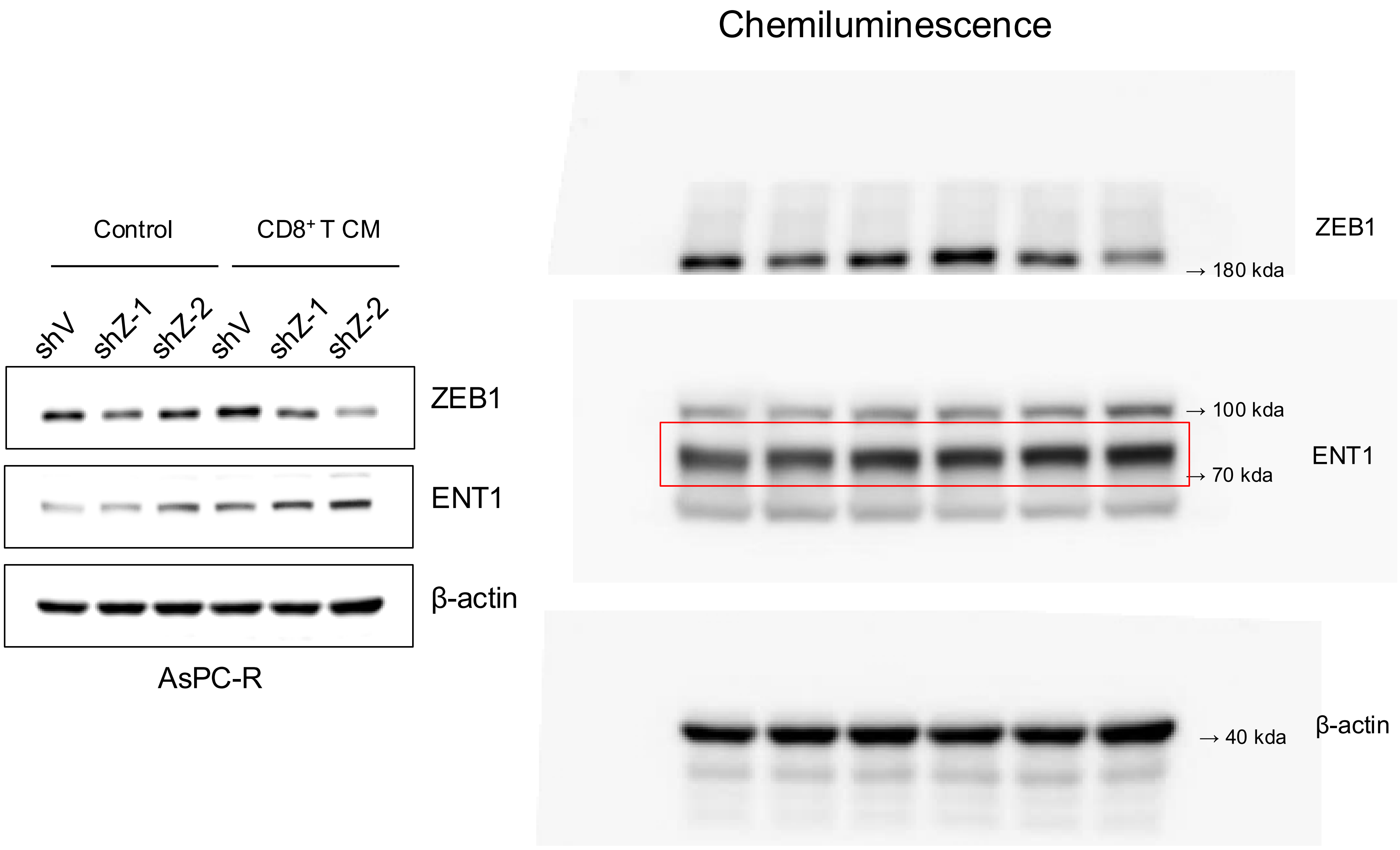
Chemiluminescence
Control
CD8+ T CM
shV
shZ-1
shZ-2
shV
shZ-1
shZ-2
ZEB1
ENT1
β-actin
AsPC-R
ZEB1
→ 180 kda
→ 100 kda
ENT1
→ 70 kda
β-actin
→ 40 kda

Figure 3K.

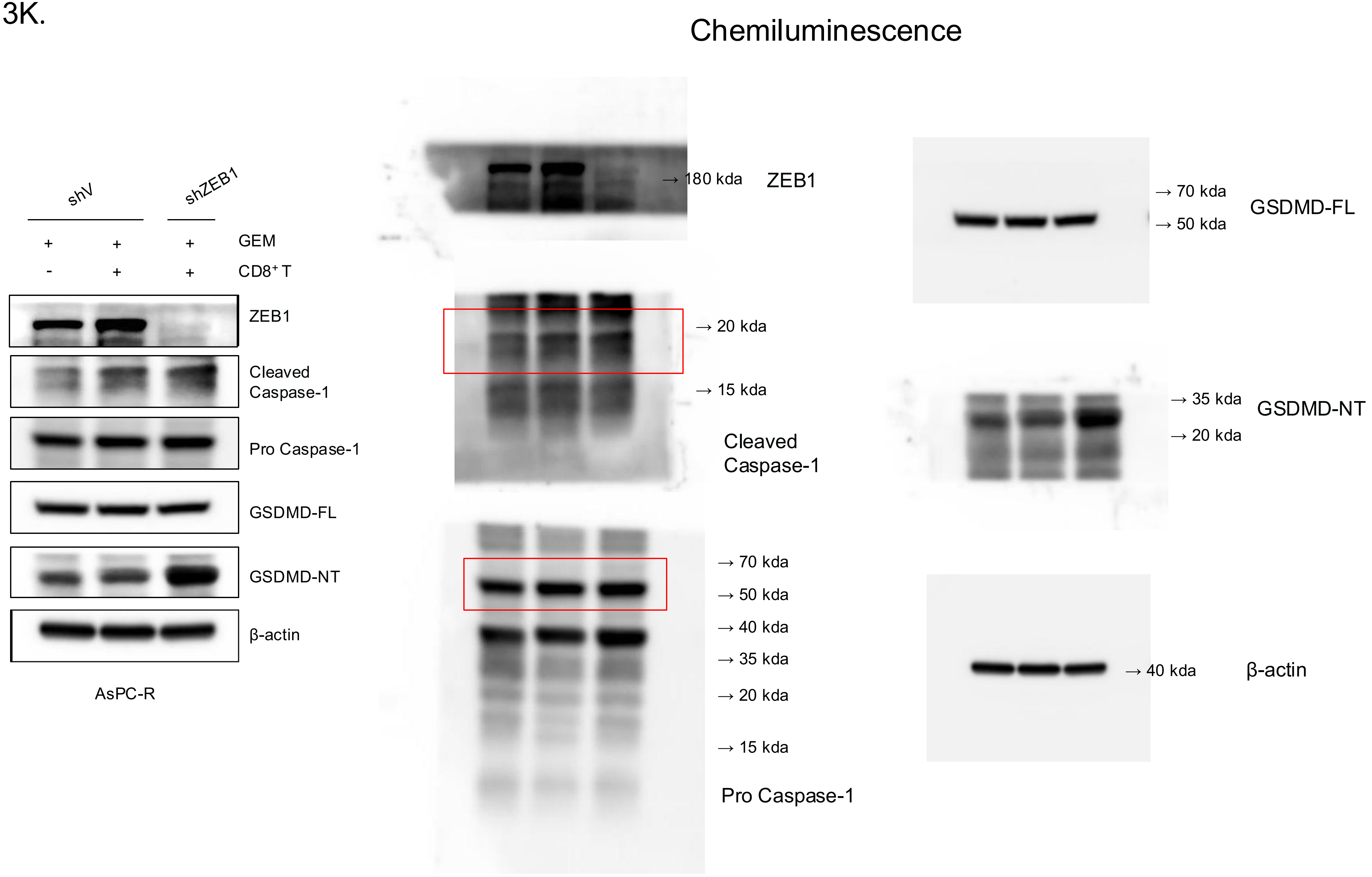

Chemiluminescence
shV
shZEB1
GEM
CD8⁺ T
ZEB1
Cleaved Caspase-1
Pro Caspase-1
GSDMD-FL
GSDMD-NT
β-actin
AsPC-R
→ 180 kda
→ 20 kda
→ 15 kda
→ 70 kda
→ 50 kda
→ 40 kda
→ 35 kda
→ 20 kda
→ 15 kda
→ 70 kda
→ 50 kda
→ 35 kda
→ 20 kda
→ 40 kda

Figure S1 H&I.

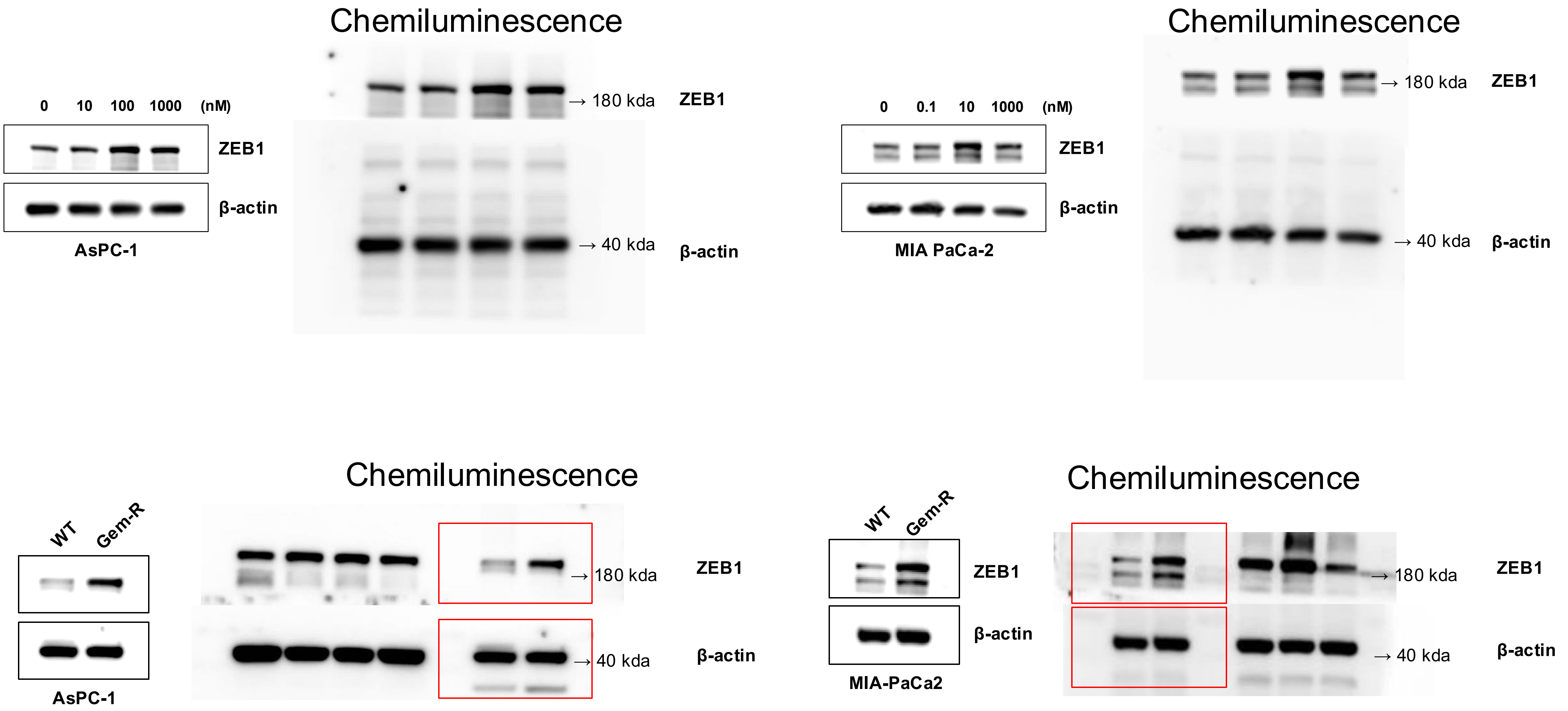

Chemiluminescence
0 10 100 1000 (nM)
ZEB1
β-actin
AsPC-1
→ 180 kda
ZEB1
→ 40 kda
β-actin
Chemiluminescence
0 0.1 10 1000 (nM)
ZEB1
β-actin
MIA PaCa-2
→ 180 kda
ZEB1
→ 40 kda
β-actin
Chemiluminescence
WT
Gem-R
AsPC-1
→ 180 kda
ZEB1
→ 40 kda
β-actin
Chemiluminescence
WT
Gem-R
ZEB1
β-actin
MIA-PaCa2
→ 180 kda
ZEB1
→ 40 kda
β-actin

Figure S1 N.

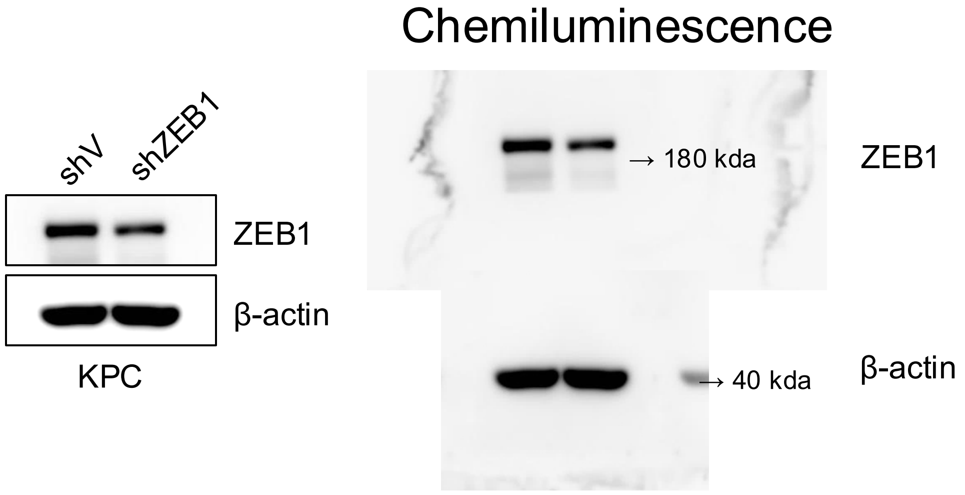

Figure S3 E.

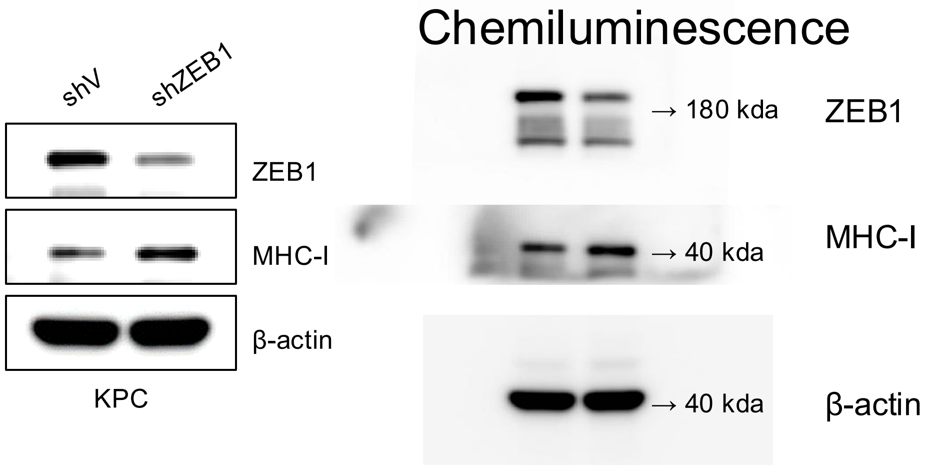

Figure S3 I.

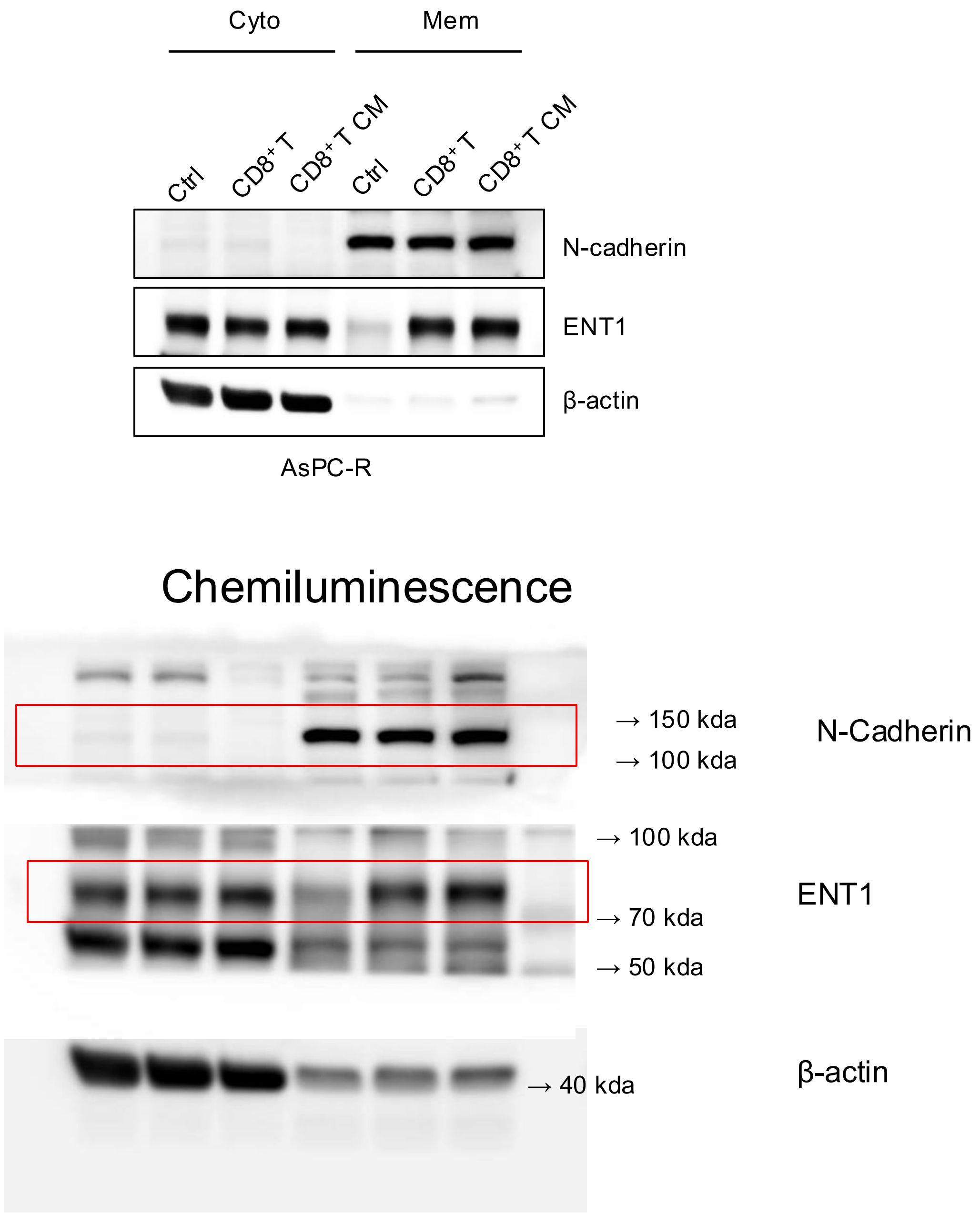

Figure S4 L.

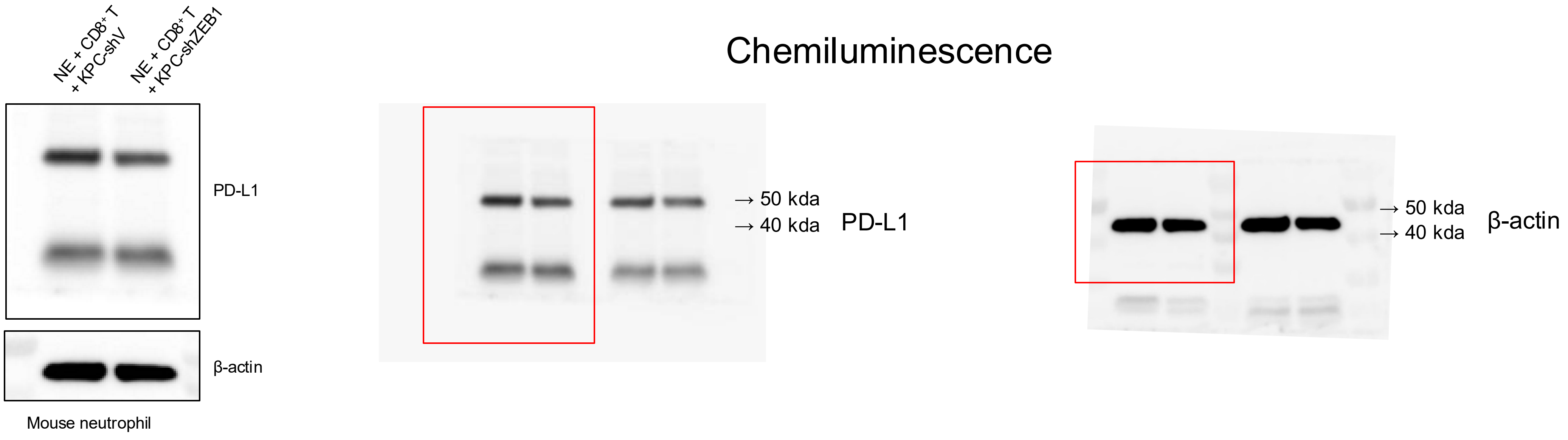

Figure S5 E.

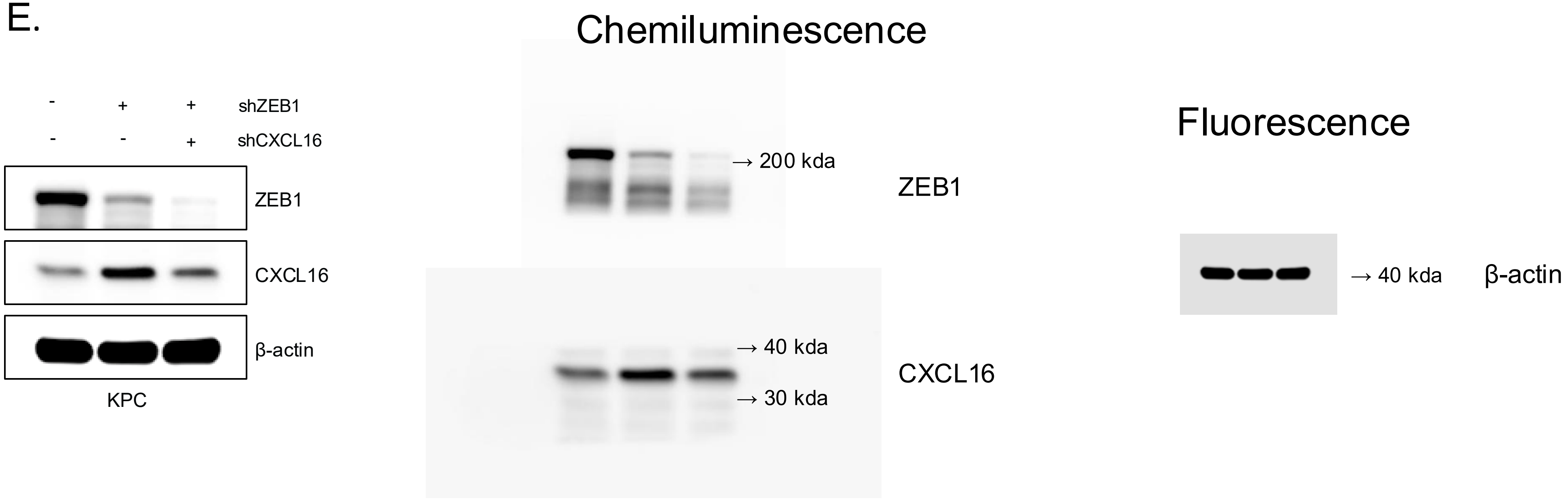

Figure S7 A.

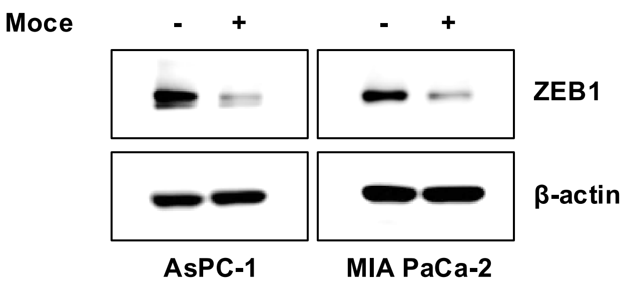

Chemiluminescence

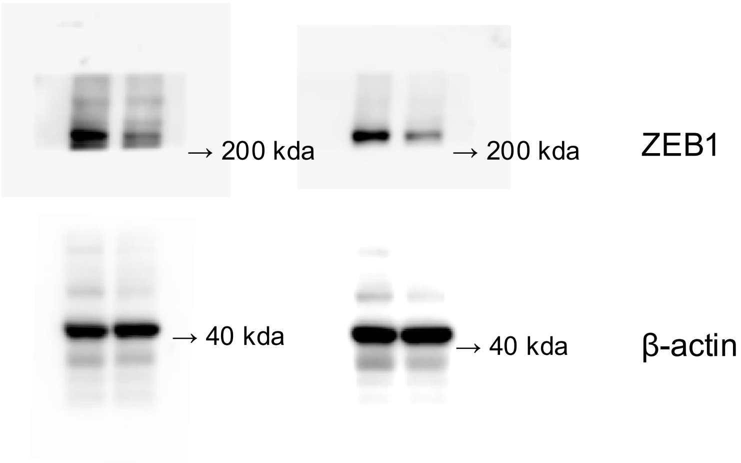

Figure S7 G.

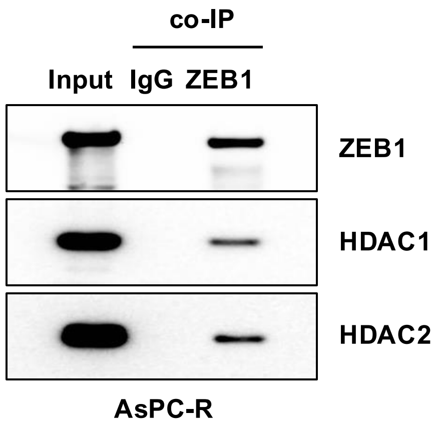

Chemiluminescence

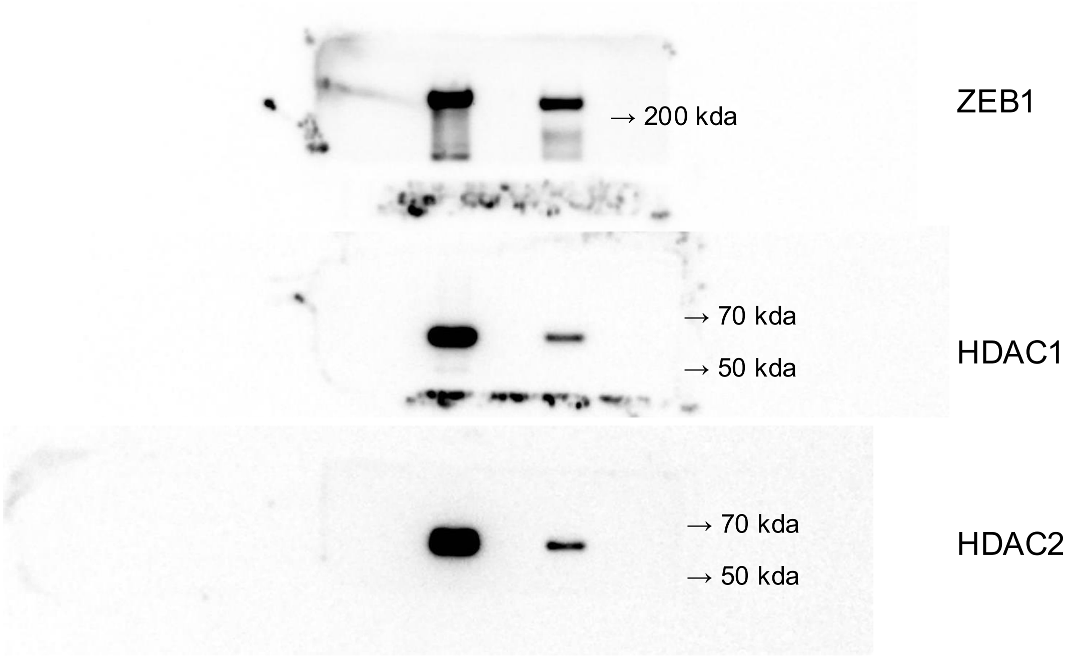

Figure S7 H.

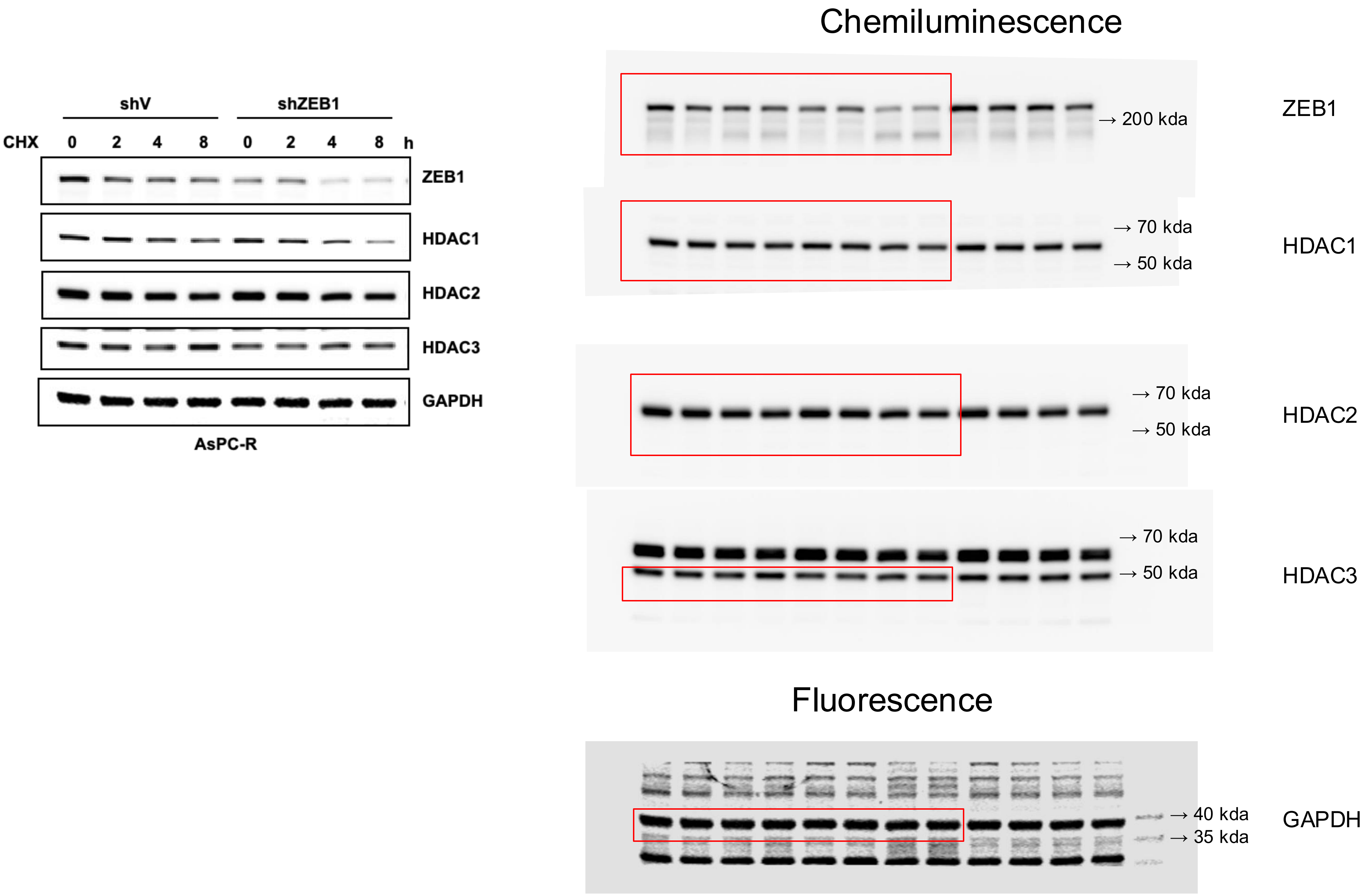
shV
shZEB1
CHX
0
2
4
8
0
2
4
8
h
ZEB1
HDAC1
HDAC2
HDAC3
GAPDH
AsPC-R
Chemiluminescence
→ 200 kda
ZEB1
→ 70 kda
→ 50 kda
HDAC1
→ 70 kda
→ 50 kda
HDAC2
→ 70 kda
→ 50 kda
HDAC3
Fluorescence
→ 40 kda
→ 35 kda
GAPDH
